# Low-energy nanoemulsions as carriers for red raspberry seed oil: Formulation approach based on Raman spectroscopy and textural analysis, physicochemical properties, stability and *in vitro* antioxidant/ biological activity

**DOI:** 10.1371/journal.pone.0230993

**Published:** 2020-04-16

**Authors:** Ana Gledovic, Aleksandra Janosevic Lezaic, Veljko Krstonosic, Jelena Djokovic, Ines Nikolic, Danica Bajuk-Bogdanovic, Jelena Antic Stankovic, Danijela Randjelovic, Sanela M. Savic, Mila Filipovic, Slobodanka Tamburic, Snezana D. Savic

**Affiliations:** 1 Department of Pharmaceutical Technology and Cosmetology, Faculty of Pharmacy, University of Belgrade, Belgrade, Serbia; 2 Department of Physical Chemistry and Instrumental Methods, Faculty of Pharmacy, University of Belgrade, Belgrade, Serbia; 3 Department of Pharmacy, Faculty of Medicine, University of Novi Sad, Novi Sad, Serbia; 4 Faculty of Physical Chemistry, University of Belgrade, Belgrade, Serbia; 5 Department of Microbiology and Immunology, Faculty of Pharmacy, University of Belgrade, Belgrade, Serbia; 6 Department of Microelectronic Technologies, Institute of Chemistry, Technology and Metallurgy, University of Belgrade, Belgrade, Serbia; 7 DCP Hemigal, Leskovac, Serbia; 8 Higher Education School of Professional Health Studies, Belgrade, Serbia; 9 London College of Fashion, University of the Arts London, London, United Kingdom; Northeastern University, UNITED STATES

## Abstract

Considering a growing demand for medicinal/cosmetic products with natural actives, this study focuses on the low-energy nanoemulsions (LE-NEs) prepared via the Phase inversion composition (PIC) method at room temperature as potential carriers for natural oil. Four different red raspberry seed oils (ROs) were tested, as follows: cold-pressed vs. CO_2_-extracted, organic vs. non-organic, refined vs. unrefined. The oil phase was optimized with Tocopheryl acetate and Isostearyl isostearate, while water phase was adjusted with either glycerol or an antioxidant hydro-glycolic extract. This study has used a combined approach to formulation development, employing both conventional methods (pseudo-ternary phase diagram − PTPD, electrical conductivity, particle size measurements, microscopical analysis, and rheological measurements) and the methods novel to this area, such as textural analysis and Raman spectroscopy. Raman spectroscopy has detected fine differences in chemical composition among ROs, and it detected the interactions within nanoemulsions. It was shown that the cold-pressed, unrefined, organic grade oil (RO2) with 6.62% saturated fatty acids and 92.25% unsaturated fatty acids, was optimal for the LE-NEs. Textural analysis confirmed the existence of cubic gel-like phase as a crucial step in the formation of stable RO2-loaded LE-NEs, with droplets in the narrow nano-range (125 to 135 nm; PDI ≤ 0.1). The DPPH test in methanol and ABTS in aqueous medium have revealed a synergistic free radical scavenging effect between lipophilic and hydrophilic antioxidants in LE-NEs. The nanoemulsion carrier has improved the biological effect of raw materials on HeLa cervical adenocarcinoma cells, while exhibiting good safety profile, as confirmed on MRC-5 normal human lung fibroblasts. Overall, this study has shown that low-energy nanoemulsions present very promising carriers for topical delivery of natural bioactives. Raman spectroscopy and textural analysis have proven to be a useful addition to the arsenal of methods used in the formulation and characterization of nanoemulsion systems.

## Introduction

In the era of the growing popularity of natural consumer products berry fruits are recognized as important sources of vitamins, minerals, antioxidants, polyunsaturated fatty acids, anti-carcinogenic compounds, UV-protective phyto-pigments and fibers [1–4]. Various types of berry fruit extracts have been used in topical formulations for dermatological and cosmetic purposes as protective, repairing and antioxidant actives [2,5]. Red raspberry (*Rubus idaeus*) fruit is a valuable source of skin vitalizing compounds. For example, its lipophilic seed oil extract is a rich source of anti-inflammatory essential polyunsaturated fatty acids − PUFAs (linoleic acid, C18:2 ω6, α-linolenic acid C18:3 ω3), and monounsaturated acids − MUFAs (oleic acid, C18:1 ω9), antioxidants (tocopherols and tocotrienols), and UV-protective carotenoids (yellow pigments) [1–4]. Moreover, hydro-glycolic extracts made from whole raspberry fruit contain fruit acids (citric, malic), sugars (fructose, inositol, sucrose) and antioxidants: tannins (polyphenols), vitamin C, and anthocyanins (red pigments) [6–8]. Another promising, but under-researched, raw material unexploited for topical application is French oak fruit (acorn) extract, a polyphenol-rich extract with the high antioxidant performance [9–11].

It is known that natural extracts represent complex mixtures of bioactive molecules that exhibit naturally occurring differences in their composition, due to the variability in the plant material, weather conditions, soil and extraction procedures [3,4,8]. These extracts are available in the market in different grades (e.g. organic or non-organic, refined or unrefined, cold-pressed or CO_2_-extracted seed oils). Consequently, it can be very difficult to predict their behavior, especially when incorporated into complex systems like colloidal carriers.

Nanocarriers, in particular nanoemulsions, have been a subject of extensive research in the last two decades, specifically as prospective delivery systems in pharmaceutical and cosmetic formulation [12–14]. Nanoemulsions are defined as kinetically stable mixtures of two immiscible liquids, with one liquid phase dispersed in the other, forming spherical droplets of 10 to 300 nm in diameter [12,15,16]. Compared to classical macroemulsions, nanoemulsions have smaller particle sizes and show high Brownian motion, which ensures better stability regarding gravity-induced phenomena (creaming and sedimentation). Unlike microemulsions, nanoemulsions are thermodynamically unstable, but they can have a long shelf life providing that Ostwald ripening, flocculation and oil phase transfer are prevented by careful choice of ingredients [12,15–17]. Nanoemulsions provide many other benefits desirable for skincare applications: (i) pleasant visual appearance (transparency, translucency or milky white color, with characteristic bluish shine) and good sensorial attributes suitable for skincare applications; (ii) better skin hydration and enhanced penetration of active substances due to homogenous and compact film of nano-droplets on the skin surface; (iii) modified release of cosmetic actives and stabilization of delicate ingredients [13,15]. Moreover, O/W type of nanoemulsions can be diluted with water without jeopardizing their structure, and their tunable rheological properties make them suitable for various dermo(cosmetic) formulations [13–15].

Due to sustainability concerns, low-energy (LE) methods are gaining popularity over conventional high-energy production methods. In the LE process, nanoemulsions is formed due to the chemical energy released from carefully selected ingredients, once they are mixed in a specific way. They are particularly suitable for the production of nanoemulsion carriers with shear- and thermo-sensitive natural ingredients, such as seed oils and fruit extracts [13–16,16,18].

This optimization study comprises three main parts:

Firstly, the screening/preformulation phase included the development of Polysorbate 80 based LE-NEs using the low-energy Phase Inversion Composition (PIC) method at room temperature. The pseudo-ternary phase diagram (PTPD) study was employed to assess the influence of four different red raspberry seed oils with very similar declared composition (Table 1) on LE-NE formation and properties. Oil phase was then optimized by employing Tocopheryl acetate and/or Isostearyl isostearate. Following that, the water phase was adjusted by adding either co-solvent glycerol or one of the antioxidant hydro-glycolic fruit extracts: from red raspberry fruit (RE) or French oak fruit (FE).

**Table 1:** Main characteristics and key ingredients of different red raspberry seed oils (ROs) as the main components of the low-energy nanoemulsion oil phase.

	Red raspberry seed oils			
	RO1	RO2	RO3	RO4
**Oil type**	Cold-pressed	Cold-pressed	CO_2_-extracted	CO_2_-extracted
	Non-organic	Organic	Non-organic	Organic
	Refined	Unrefined	Unrefined	Unrefined
**Visual appearance**	Pale yellow	Orange-yellow	Pale green-yellow	Golden yellow
**Fatty acid composition** (as a percent of total)				
C16:0 Palmitic (2–6%)	3.12%	5.11%	2.2%	2.2%
C18:0 Stearic (≤ 3.0%)	0.92%	1.51%	0.91%	0.89%
C18:1 ω9 Oleic (8–14%)	12.10%	12.2%	12.6%	12.7%
C18:2 ω6 Linoleic (50–62%)	51.94%	58.05%	59.1%	59.0%
C18:3 ω3 Linolenic (21–36%)	30.01%	22.0%	24.4%	24.1%
Total of saturated acids	4.04%	6.62%	3.11%	3.09%
Total of unsaturated acids	94.05%	92.25%	96.1%	95.8%
**ω6 : ω3 ratio**	1.73	2.64	2.42	2.45
**Sum of Tocopherols** (as alpha-Tocopherol)	n.s.	n.s.	0.12%	0.28%
**Sum of Tocotrienols**	n.s.	n.s.	0.03%	0.03%

*n.s.—not stated.

Secondly, Raman spectroscopy was performed to detect chemical differences among various red raspberry seed oils (ROs) as well as their respective LE-NEs. This advanced technique can be used to determine the degree of unsaturation in vegetable oils, e.g. the saturated vs. unsaturated fatty acid content [19,20]. It was recently reported that Raman spectroscopy can also be used to detect structural changes when oils and curcumin [21] or proteins [22] are incorporated into nanodroplets, as well as to study interactions among nanoemulsion components. Textural and rheological investigations were employed as additional techniques to determine the nature of transient liquid crystalline phases and the effect of oil/ water phase variations on the LE-NE formation and stability [23,24].

Finally, the optimized nanoemulsion formulations with lipophilic and/or hydrophilic antioxidant extracts were analyzed *in vitro* in terms of their antioxidant potential and biological activity before and after nanoemulsification. For the purpose of safety evaluation, the normal human fibroblasts (MRC-5) have been used, while the anti-proliferative effect on HeLa and Fem-X cells was investigated as a screening study of potential anticancer effects.

## Materials and methods

### Materials

Raspberry seed oils: Four different red raspberry seed oils (ROs) were received as free samples for research purposes (Table 1). Two were cold-pressed seed oils: RO1−refined, non-organic (Seatons, East Yorkshire, United Kingdom) and RO2−unrefined, organic (produced by Aromaaz International, New Delhi, India, for domestic brand Eterra/company Terra Co, Novi Sad, Serbia), both with the same INCI name: Rubus idaeus seed oil. The other two were CO_2_-extracted seed oil extracts: RO3−non-organic and RO4−organic, (both from Flavex Naturextrakte GmbH, Rehlingen, Germany), INCI name: Rubus idaeus seed (Raspberry) extract and Rosmarinus officinalis (Rosemary) leaf extract since they contained up to 0.1% of the rosemary leaf extract as an antioxidant.

Other oil phase components: CRODAMOL® ISIS (INCI: Isostearyl isostearate) was received as a free sample from CRODA, East Yorkshire, United Kingdom, and alpha-Tocopheryl acetate was produced by FAGRON, Trikala, Greece.

Water phase: Red raspberry fruit extract − Fruitliquid® raspberry (INCI: Water, Propylene glycol, Rubus idaeus fruit extract) and French oak fruit extract − Phytessence® French oak (INCI: Aqua, Glycerin, Quercus petraea fruit extract) were received as free samples from CRODAROM, Chanac, France. Glycerol was produced by Fisher Chemical, Waltham, ME, USA. Ultra-purified water was obtained with GenPure apparatus (TKA Wasseranfbereitungssysteme GmbH, Germany).

Surfactant: Non-ionic surfactant Polyoxyethylene-20 sorbitan monooleate (INCI: Polysorbate 80) was produced by Sigma-Aldrich Laborchemikalien GmbH, Seelze, Germany.

Reagents for antioxidant assays: 2,2-Diphenyl-1-picrylhydrazyl (DPPH), 2,2'-Azino-bis (3-ethylbenzothiazoline-6-sulfonic acid) diammonium salt (ABTS), potassium persulfate, (±)-6-Hydroxy-2,5,7,8-tetramethylchromane-2-carboxylic acid (Trolox) and methanol (HPLC grade) were produced by Sigma-Aldrich. Phosphate buffered saline (PCS buffer) of pH value 7.4 was prepared fresh before the ABTS assay.

### The screening/preformulation phase

#### PTPD study with different red raspberry seed oils

Identification of low-energy nanoemulsion (LE-NE) and the transient liquid crystalline (LC) regions was performed via PTPD study, along water dilution lines (90:10, 80:20, 70:30, 60:40, 50:50, and 40:60) with four different red raspberry seed oils (ROs) and surfactant Polysorbate 80 (P80). The samples were prepared using PIC method at room temperature, by stepwise addition of water with continuous vortex mixing (at 1300 rpm) to previously prepared surfactant-oil (SO) mixtures (2 minutes, at 1600 rpm). It should be noted that, until the semi-solid transient LC gel-like phases were passed (at about 20 to 40 wt% water), hand mixing with glass laboratory sticks was employed. Short homogenization of samples was performed by vortex mixing for 2 minutes at 1300 rpm to obtain oil in water (O/W) LE-NEs at about 70 to 80 wt% water.

*Particle size measurements*. Particle size distributions were measured using a dynamic light scattering (DLS) device (Zeta Sizer Nano ZS, Malvern Instruments, Malvern, UK) from LE-NE samples freshly diluted with ultra-purified water (1:100 v/v for milky white or 1:10 v/v for transparent samples) to avoid multiple light scattering. Measurements were performed 24 to 48h after preparation, in triplicate, and after 30 to 45 days of storage at room temperature. Additionally, the LE-NEs acceptable according to DLS measurements and microscopical analysis were further checked after 45 days of storage at room temperature, using laser diffraction (LD) instrument (Beckman Coulter LS 13320, universal liquid module) to exclude the presence of bigger particles (aggregates).

#### Oil phase optimization–influence of Isostearyl isostearate and Tocopheryl acetate

Different red raspberry seed oils were mixed in various ratios with emollient ester Isostearyl isostearate and/or antioxidant ester alpha-Tocopheryl acetate to assess their influence on LE-NE formation and stability. Total oil phase concentration was 10 wt% of LE-NE. Clear or slightly opalescent SO mixtures were considered indicators of good compatibility among selected ingredients.

#### Water phase optimization − influence of glycerol and antioxidant hydro-glycolic fruit extracts

Glycerol, as a potential co-solvent and stabilizer in LE-NE formulations, or antioxidant hydro-glycolic fruit extracts prepared from red raspberry–RE or French oak–FE fruit, were added to ultra-purified water in order to investigate their influence on LE-NE formation and stability. For example: 5, 10 or 15 wt% solutions of glycerol and 5 or 10 wt% solutions of RE and FE extracts, respectively, were used instead of ultra-purified water. Total water phase content was set to 80 wt% of the LE-NE.

### Formulation study of red raspberry seed oil-loaded LE-NEs: Physicochemical properties, stability and *in vitro* antioxidant activity

Firstly, Raman spectra of four different red raspberry seed oils (ROs), surfactant (P80), glycerol and RO-loaded LE-NEs were analyzed in order to evaluate the differences among ROs and the corresponding LE-NEs. Based on the preformulation study and the Raman investigations, several characteristic preliminary stable samples of transient LC gel-like phases and corresponding LE-NEs (Table 2) were prepared with RO2 (the organic, cold-pressed, unrefined seed oil) and thorough microscopical/ textural/ rheological investigations were performed to elucidate the influence of the main LE-NE components on PIC LE-NE formation, properties and stability. Finally, antioxidant performance and storage stability (at 4, 25 and 40°C) were investigated in samples containing lipophilic antioxidants from RO2, and the RO2-loaded LE-NEs containing additional antioxidant extracts (RE, FE) in the LE-NE water phase.

**Table 2:** Final composition (wt%) of the RO2-loaded gel-like phases and low-energy nanoemulsions investigated in the formulation studies.

	P80	RO2	ISIS	TA	GLY	RE	FE	Water, purified
**Gel-like phases**								
**G1**	35	31.5	–	3.5	3	–	–	27
**G2**	35	15.75	15.75	3.5	3	–	–	27
**G3**	35	31.5	–	3.5	–	1.5	–	28.5
**G4**	35	31.5	–	3.5	–	–	1.5	28.5
**Nanoemulsions**								
**F1**	10	9	–	1	8	–	–	72
**F2**	10	4.5	4.5	1	8	–	–	72
**F3**	10	9	–	1	–	4	–	76
**F4**	10	9	–	1	–	–	4	76

* P80-Polysorbate 80; RO2-red raspberry seed oil, cold-pressed, organic, unrefined; ISIS-Isostearyl isostearate; TA-Tocopheryl acetate; GLY-Glycerol; RE-Red raspberry hydro-glycolic fruit extract; FE- French oak hydro-glycolic fruit extract.

#### Raman spectroscopy of red raspberry seed oils and corresponding LE-NEs

Raman spectra of raw materials (ROs, glycerol, P80) and the LE-NEs prepared with these components were recorded with a DXR Raman microscope (Thermo Fisher Scientific, Medison, Wisconsin, USA), equipped with a research optical microscope and a CCD detector, using a frequency-stabilized single mode diode laser with an excitation wavelength of 780 nm. For more details, please consult the Supporting information.

#### Microscopical analysis

*Optical (polarization) microscopy*. Optical (polarization) microscopy was employed for the screening of LC gel phases during the PTPD study with four different ROs, and for the inspection of RO2-loaded LC gel-like phases of varied composition (Table 2) as well as to investigate the presence of micro-range structures/aggregates in LE-NEs. All samples were investigated undiluted, at different magnifications (100x, 200x and 400x) using Motic digital DMB3-22ASC microscope (Motic GmbH, Germany), with or without polarized light lens, equipped with Motic Images Plus v.2.0 software.

*Atomic force microscopy (AFM)*. AFM was employed as a direct technique to investigate the microstructure and topography of LE-NE samples prepared with RO2 (Table 2) and to confirm the results obtained by DLS and LD measurements. The AFM model employed was AutoProbe CP-Research SPM (TM Microscopes-Bruker, Germany). For more details on the experimental protocol, please consult the Supporting information.

#### Electrical conductivity and pH value measurements

The electrical conductivity of the undiluted RO2-loaded LE-NEs and characteristic transient LC gel-like phases along selected dilution lines was measured using SENSION+ EC71 apparatus (HACH, Loveland, Colorado, USA). The pH value was measured with undiluted LE-NE samples using HI9321 microprocessor pH meter (Hanna Instruments Inc., Ann Arbor, Michigan, USA). The measurements were performed in triplicate at 25±2°C according to the testing protocol.

#### Rheological and textural analysis

The range of RO2-loaded preliminary stable samples (Table 2) was used as a model to gain further insight into the structure of transient gel-like LC phases detected during PIC LE-NE formation at 50:50 surfactant-to-oil ratio (SOR) and to investigate the link between them and the corresponding LE-NEs. Rheological tests were carried out by HAAKE Mars rheometer (Thermo Electron Corporation, Karlsruhe, Germany) at a constant temperature of 25 ± 0.1°C. A minimum of three measurements was performed for each tested material.

*Oscillatory rheology tests of gel-like phases*. Viscoelastic properties of gel-like LC phases were analyzed through amplitude and frequency sweep tests with parallel plates PP35 sensor. Amplitude sweep tests were done to detect the linear viscoelastic region (LVR) from the plot storage G’ and loss G” moduli versus shear stress τ (0.1–50 Pa) at a constant frequency (1 Hz). Frequency sweep tests were performed from 0.1 to 10 Hz at constant shear stress (1 Pa) to determine the variation of the complex viscosity (η*), storage (elastic) shear modulus (G’) and loss (viscous) shear modulus (G”).

*Textural analysis of gel-like phases*. Textural characteristics (firmness, consistency, adhesiveness) of gel-like phases 7 days after preparation were measured by a TA.XT-Plus Texture analyzer (Stable Micro Systems, UK) using a P/5 cylinder stainless probe with 5 mm diameter. The experiment was performed at the pre-test speed of 1 mm/s, the test speed of 2 mm/s and the post-test speed of 2 mm/s, and the compression distance of 5 mm. Textural analyses were conducted at 23 ± 0.1°C in three replicates per batch.

*Continuous flow tests of LE-NEs*. Continuous flow (hysteresis loop) tests were carried out with a cylinder DG 41 titanium sensor. The LE-NE samples were first exposed to the increasing shear rate from 0.5 to 100 s^-1^, then sheared for 30 s at 100 s^-1^ and finally exposed to a decreasing shear rate back to 0.5 s^-1^, the up and down curve each taking 100 s. It was not possible to perform continuous flow tests for gel-like phases since they exhibited the Weissenberg effect.

#### *In vitro* antioxidant activity and storage stability

*ABTS assay*. The ABTS assay was performed as described by de Souza et al. [7] with small modifications. Trolox solutions in PCS buffer were used as standard compounds to validate the method. Details of the testing protocol are presented in the Supporting information section. Results were expressed as %INH (Percentage of ABTS^+^ inhibition) according to the equation: % Inhibition of ABTS^+^ radical = [(A_ABTS_^+^—A_sample_) / A_ABTS_^+^] x 100, where A_ABTS+_ is the absorbance of ABTS^+^ solution (0.7) and A_sample_ is the absorbance of samples, after 6 minutes of reaction time.

*DPPH assay*.The DPPH assay was performed according to the method described by Rebolleda et al. [25], with small modifications. Different concentrations of Trolox solutions in methanol were used as a standard antioxidant to validate the method. Details of the testing protocol are presented in the Supporting information section. Results were expressed as %INH (Percentage of DPPH inhibition) according to the equation: % Inhibition of DPPH radical = [(A_DPPH_—A_sample_) / A_DPPH_] x 100, where A_DPPH_ is the absorbance of DPPH standard sample (5ml of DPPH standard solution mixed with 5 ml methanol) and A_sample_ is the absorbance of samples after 30 minutes.

*Storage stability*. Storage stability assessment (45 days stability study at 4, 25 and 40°C) of the selected RO2-loaded LE-NEs (Table 2) was performed using pH, electrical conductivity and DLS measurements. All measurements were done in triplicate at room temperature, 24 to 48 hours after preparation and upon storage of samples at different temperatures.

#### *In vitro* biological activity

*Cell cultures*. Three cell cultures were used: normal human lung fibroblasts (MRC-5) and tumor cells–human cervical adenocarcinoma (HeLa) and human malignant melanoma (Fem-X). The cells were maintained in complete nutrient medium RPMI-1640 at 37°C in a humidified atmosphere with 5% CO_2_. All cell lines were obtained from American Type Culture Collection (Manassas, VA, USA). For all of the cells used, the nutrient medium was RPMI 1640 (Sigma-Aldrich, St. Louis, USA), supplemented to final concentration with L-glutamine (3 mM), streptomycin (100 mg/mL), penicillin (100 IU/mL), and fetal bovine serum (10%; heat-inactivated at 56°C for inactivation of cholinesterases, system complement and HEPES (25 mM)), adjusted to pH 7.2 (bicarbonate solution).

*Preparation of stock solutions of raw materials and nanoemulsions*. The stock solution of the red raspberry seed oil (RO2) was prepared in dimethyl sulfoxide (DMSO), afterward, it was diluted in the nutrient medium so that the final DMSO concentration is below 0.1% i.e. 600 μg/ml RO2. RO2-loaded nanoemulsions F1, F3 and F4 (for composition see Table 2) were diluted directly with the medium to match the tested concentrations of pure RO2. The placebo nanoemulsion (without RO2, hydro-glycolic fruit extracts of Red raspberry–RE, or French oak–FE) was diluted in the same manner to ensure appropriate experimental protocol. RE and FE were diluted directly with the medium to match their concentration in samples F3 and F4, respectively. All samples were filtered through Millipore filters (0.22 μm) and diluted for use in the nutrient medium to the working concentrations.

*Treatment of the cell lines*. The MRC-5 (5x10^3^ cells per well), HeLa (2x10^3^ cells per well) and Fem-X (5x10^3^ cells per well) cells were seeded into 96-well microtiter plates, and 24 h later, after cell adherence, five different concentrations of the test samples were added to the wells. The test concentration range of red raspberry seed oil (per se or in the nanoemulsion) and RE, FE extracts was 12.5, 25, 50, 100 μg/ml and 200 μg/ml, and the corresponding dilutions of the placebo nanoemulsion (0.222, 0.111, 0.056, 0.028, 0.014 v/v %) were also investigated. In the control wells, only nutrient medium was added to the cells.

*Determination of target-cell survival*. Cell viability was determined by the MTT test, 48h after the addition of the samples. 3-(4,5-dimethylthiazol-2-yl)-2,5-diphenyl tetrazolium bromide–MTT (Sigma-Aldrich, St. Louis, USA) was dissolved in phosphate buffered saline (PBS), pH 7.2 (5 mg/mL), and filtered through Millipore filters (0.22 μm) before use. Briefly, 10 μL MTT solution (5 mg/mL in the PBS) was added to each well. The samples were incubated for an additional 4 h at 37°C, in 5% CO2 and humidified atmosphere. Then, 100 μL 10% sodium dodecylsulfate–SDS (Sigma-Aldrich, St. Louis, USA) was added to each of the wells, and the absorbance of the cell medium from each well was measured at 570 nm the next day. Measurements were performed using Multiskan^™^ FC Microplate Photometer (Thermo Scientific, USA).

The cell viability (%) was calculated from the measured absorbance at 570 nm of each of the samples containing cells grown in the presence of the test compounds divided by the absorbance of the control sample (the absorbance of cells grown in nutrient medium only), after the subtraction of the blank sample absorbance. The IC50 values (the concentration of compound which decreased the survival of treated cells by 50%) were determined from the graph by numerical analysis of the obtained data. Three independent experiments were performed, each in triplicate, and results were presented as mean values ± SD.

### Statistical analysis

One-way ANOVA with Tukey post hoc test was performed for 3 and more normally distributed data groups (OriginPro8.5), or two-way ANOVA when the two factors varied at the same time (OriginPro8.5, SPPS statistics 20), with the appropriate post hoc test (Tukey or Fisher LSD). For small data samples or for non-normally distributed data, a non-parametric Kruskal Wallis ANOVA was used, with pairwise comparisons (IBM SPSS Statistics 20). The significance level was set to p < 0.05.

## Results and discussion

### The screening/preformulation phase

#### PTPD study with different red raspberry seed oils

In the pharmaceutical and cosmetic industry, it is common to have ingredients with the same chemical name offered by different suppliers. As it was previously pointed out [3,4,8], there are many specialty ingredients, particularly those of natural origin, available in different grades (e.g. organic or non-organic, refined or unrefined seed oils), which is a challenging situation in the formulation/production of complex systems that rely on physicochemical compatibility of all ingredients, such as low-energy nanoemulsions (LE-NEs).

Among the available low-energy methods to obtain nanoemulsions, Phase inversion composition (PIC) is one of the most frequently applied techniques. Starting from the surfactant-oil (SO) mixtures, and adding water in the stepwise manner, the system passes through several phases: (i) W/O emulsion, (ii) LC phase/ microemulsion (ME)/ O/W/O multiple emulsion, (iii) O/W nanoemulsion [15,18,26]. In this study, the PIC method performed at room temperature was chosen to protect the sensitive active ingredients present in red raspberry seed oil (PUFAs, tocopherols, tocotrienols, and carotenoids). Polysorbate 80 (P80) is commonly used in the pharmaceutical and cosmetic industry as non-ionic surfactant and solubilizer, due to its proven effectiveness and acceptable safety profile. Moreover, as a small molecular weight surfactant with high HLB value (HLB~15.0), P80 is suitable for generating low-energy O/W NEs, MEs and various LC structures [26–28].

**Fig 1:**
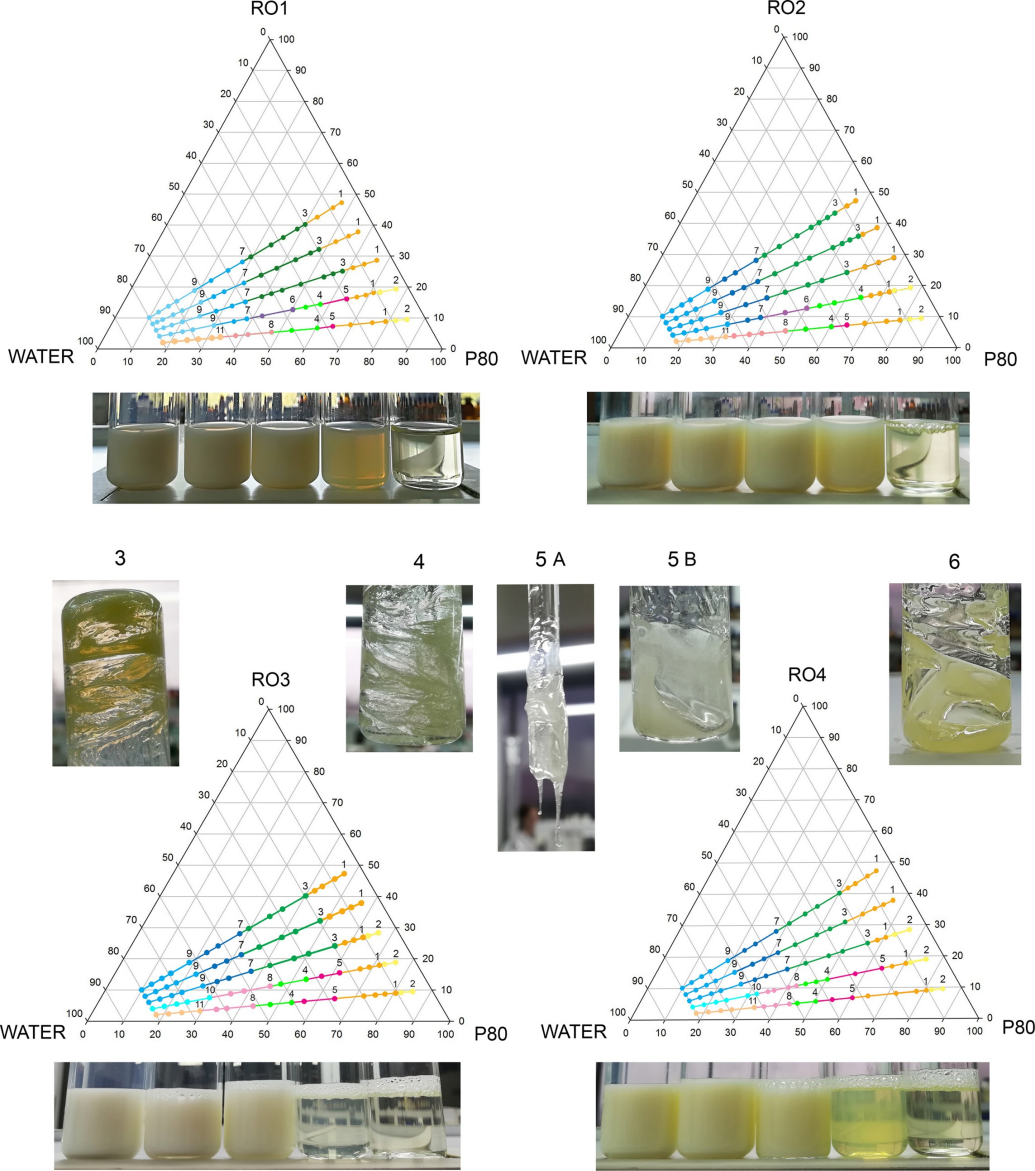
PTPD study of different red raspberry seed oils (RO1 – RO4)/ Polysorbate 80/ ultra-purified water (80 wt%) systems with the visual appearance of low-energy nanoemulsions (LE-NEs), microemulsions (MEs) and the characteristic transient LC phases. The numbers representing the LC phases are: (3) semi-transparent, semi-solid isotropic LC gel-like phase, (4) transparent, semi-solid anisotropic LC gel-like phase, (5A, 5B) turbid, liquid, anisotropic LC phase, (6) semi-transparent, liquid, anisotropic LC phase. The numbers representing liquid emulsions are: (1) W/O transparent emulsion, (2) opalescent or turbid W/O emulsion, (7) milky white, liquid O/W emulsion, (8) semi-transparent, liquid O/W emulsion, (9) low viscosity, milky white O/W LE-NE, (10) low viscosity, transparent or slightly opalescent O/W LE-NE, (11) low viscosity, transparent O/W MEs of red raspberry seed oil.

As it is well known, surfactant-to-oil (SOR) and surfactant-to-emulsion (SER) ratios are among the most important parameters in LE-NE formation and stability [18,26]. Therefore, the first step in this research was the PTPD study (using visual and microscopy observations and the mean droplet sizes PDI measurements) with four different ROs, to detect their phase behavior i.e. to elucidate the boundaries of LC, ME and NE regions. This was very important since all these nanocarriers could be prepared from the same ingredients, by employing different SOR/SER values, at different water content. The results are presented in Fig 1.

Even though all ROs were similar regarding the declared fatty acid profiles (Table 1), their visual appearance was quite different. Consequently, the visual appearance (color and level of transparency) of the SO mixtures, but also the mean droplet sizes and PDI values of the corresponding systems (at 80 wt% water), were also different (Table 3).

**Table 3:** Visual appearance of the SO mixtures and the main characteristics of the resulting low-energy nanoemulsions/microemulsions prepared with different red raspberry seed oils (RO1 – RO4) during the PTPD study of RO/P80/Water system.

Red raspberry oil type	RO1	RO2	RO3	RO4
**SOR 50:50**				
**SO mix**	Turbid, yellow	Turbid, yellow-orange	Transparent, light-green	Turbid, golden-yellow
**Z-ave (nm)**	161.37±16.5	134.83±0.035	163.2±0.265	148.1±0.458
**PDI**	0.108±0.016	0.071±0.018	0.120±0.008	0.088±0.024
**SOR 60:40**				
**SO mix**	Opalescent, yellow	Opalescent, yellow-orange	Transparent, light-green	Turbid, golden-yellow
**Z-ave (nm)**	145.23±1.007	132.77 ± 1.159	134.43±0.577	140.1±1.179
**PDI**	0.082±0.025	0.061 ± 0.021	0.110±0.026	0.105±0.016
**SOR 70:30**				
**SO mix**	Opalescent, yellow	Opalescent, yellow-orange	Transparent, light-green	Transparent, golden-yellow
**Z-ave (nm)**	126.17±0.513	141± 4.063	137.2±1.127	139.67±2.603
**PDI**	0.049±0.020	0.067± 0.049	0.050±0.025	0.064±0.009
**SOR 80:20**				
**SO mix**	Transparent, yellow	Transparent, yellow-orange	Transparent, light-green	Transparent, golden-yellow
**Z-ave (nm)**	128.53±1.625	136.93 ± 1.332	18.64±0.256	68.64±0.450
**PDI**	0.159± 0.040	0.116 ± 0.049	0.337±0.022	0.508±0.024
**SOR 90:10**				
**SO mix**	Transparent, yellow	Transparent, yellow-orange	Transparent, light-green	Transparent, golden-yellow
**Z-ave (nm)**	19.72± 9.311	21.05±7.729	13.77±1.309	28.79±1.707
**PDI**	0.175±0.028	0.190±0.093	0.306± 0.067	0.932±0.065

Z-average droplet size (Z-ave) and polydispersity index (PDI), 24 h after preparation. The values represent the means ± standard deviation of three measurements

However, some similarities were observed: in order to obtain LE-NEs, the minimal SER value was 10, while minimal SOR value was 1.0 (50:50 ratio) for all tested oils. A very important finding was that all ROs can form LE-NEs, in a simple ternary system composed of P80/RO/Water by moderate mixing. This behavior could be linked to the specific composition of red raspberry seed oils (up to 85% PUFAs and around 12% MUFA–mostly oleic acid). It is known that isolated PUFAs, as well as natural oils containing them, can form various LC structures [23,27]. Therefore, the compatibility among the hydrophobic oleic acid tails in the P80 molecule [28] and ROs seems to be based on their structural similarities, resulting in the spontaneous formation of nanodroplets.

The statistical analysis of the simultaneous effects of SOR and the RO type on droplet sizes and PDI values implied significant differences between the LE-NEs prepared with different SOR and with the same RO, as well as among LE-NEs prepared with the same SOR but different oil type. The interactions between the oil type and SOR (presented in S1 Fig, Supporting information) were also significant (two-way ANOVA, Tukey post hoc test, p level <0.05). Therefore, it was not possible to change the raspberry oil type without an adjustment in SOR, to obtain LE-NEs with desirable characteristics (e.g. visual appearance/ particle size).

The explanation for such behavior could be found in the fine differences among the tested oils. The cold-pressed oils, especially unrefined ones are believed to retain more tocopherols, carotenoids, phenolic compounds and phytosterols than the extracted oils [3,4,8]. The mentioned ingredients, which are usually not precisely specified, could be active at the surfactant-oil interface [26]. The type of plant material is also important, since it was found that tocopherols and carotenoids can vary up to 2–3 times in red raspberries of different varieties [4]. A similar phenomenon was observed in our study: the RO3 non-organic oil and RO4 organic oil, although produced identically (by CO_2_-extraction), had different content of tocopherols (Table 1). Another possible explanation could be found in the presence of stated or not stated additives in these oils. For example, RO3 and RO4 contain ≤ 0.1% of Rosemary leaf extract (antioxidant) that could also contribute to the observed differences between these oils, while RO1/RO2 oils were without additives. Small differences in the oil saturation/unsaturation ratio (Table 1), which also depend on the oil extraction technique [3], could also play a major role in the differences observed among tested oils.

#### Oil phase optimization − influence of alpha-Tocopheryl acetate and Isostearyl isostearate on LE-NE properties

Low-energy nanoemulsions in the PTPD study contained only ROs as their oil phase, therefore inevitably all samples with lower SOR values showed some signs of instability (aggregation, creaming) after a few days/weeks of storage at room temperature. The observed order of stability was: RO2>RO1>RO3>RO4-loaded LE-NEs. Since LE-NE transparency and droplet sizes bellow 100 nm were not the imperative in this formulation development, and the aim was to create LE-NEs with the highest RO loading, all further optimization was performed using the minimal SOR value (SOR 1), while the RO content was set to 4.5 to 9 wt% of LE-NE (with total oil phase of 10 wt% of LE-NE).

In order to improve the stability of RO-loaded LE-NE formulations, ROs were mixed with lipophilic esters, which is a standard step to prevent Ostwald ripening, flocculation, coalescence or the oily phase transfer between oil droplets, as the main causes of nanoemulsion instability [15,17,18]. We hypothesized that this could also minimize the effect of the observed differences among ROs on LE-NE formation and characteristics.

Apart from its declared antioxidant effects in the formulation and on the skin [29,30], it was reported that alpha-Tocopheryl acetate (TA) can also act as a cosurfactant and reduce particle sizes in LE-NEs due to its amphiphilic nature/positioning at the oil-water interface. The other mechanism of TA action is based on its high viscosity that influences the viscosity of the oil phase and, consequently, the packing of transient phases during the LE-NE formation [26,31].

**Fig 2:**
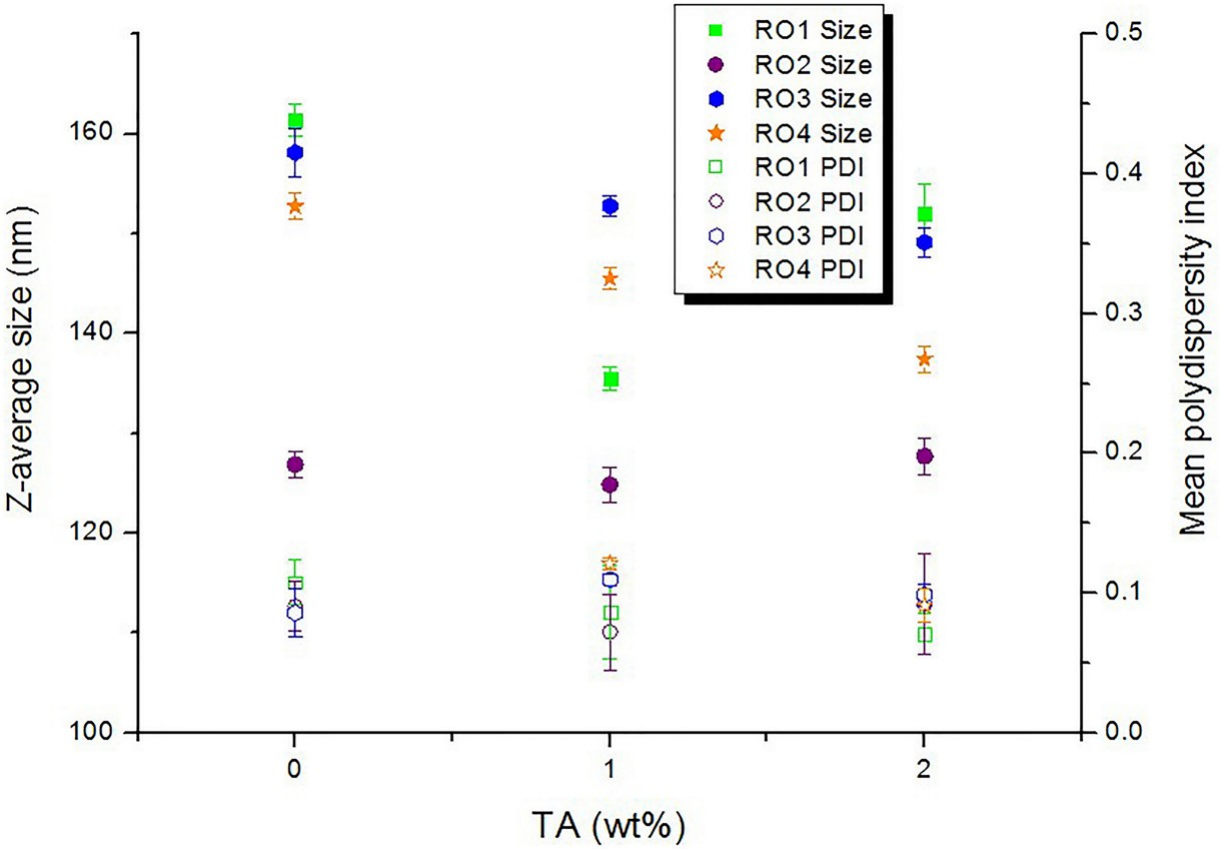
The influence of Tocopheryl acetate concentration (TA wt%), on mean droplet size (Z-average size) and mean polydispersity index of RO-loaded low-energy nanoemulsions, 24 h after preparation.

The results presented in Fig 2 indicate that the addition of 1 to 2 wt% TA to ROs significantly reduced droplet sizes of all LE-NEs, and that the optimal RO:TA ratio varied for different ROs (RO: TA ratio of 9:1 for the cold-pressed oils **−** RO1, RO2 and 8:2 for CO_2_-extracted oils − RO3, RO4) (two-way ANOVA at p< 0.05). Moreover, TA improved the stability of LE-NEs at room temperature (droplet sizes were unchanged after 45 days, and the formation of visible aggregates or creaming was inhibited in most NEs, except the RO4-loaded ones). These results are in line with other work that confirmed the stabilizing effect of TA on nanoemulsion formulations [26,31].

Another oil phase ingredient considered for additional stabilization and formulation improvement for skincare application was Isostearyl isostearate (ISIS) − a medium polarity, fully saturated, emollient ester with a branched-chain structure and of natural origin. ISIS is known to improve skin barrier function, while giving a pleasant skin feel and good spreadability to topical products [32,33]. Its use has not been explored in nanosized carrier systems, particularly in combination with natural ingredients. This study has found that ISIS can be used to create LE-NEs as a single component of the oil phase (LE-NEs loaded with 10 wt% ISIS had droplet sizes ~ 158 nm, PDI≤ 0.10, at SOR 1). When combined with RO, the optimal ISIS: RO ratio was 1 with the optimal SOR value also 1, at 10 wt% oil phase. The resulting LE-NEs had particle sizes from 130 to 170 nm and PDI value ≤ 0.1, depending on the oil type. After the addition of TA to the mix, the particle sizes did not exceed 152 nm, showing that TA had the effect of a decreasing particle size in LE-NEs prepared with mixed oil phases. LE-NEs with RO2 had the smallest particle sizes (131 nm, at RO:ISIS:TA ratio 4.5:4.5:1), followed by RO1, RO4 and RO3-loaded LE-NEs (143 nm, 145 nm, and 151 nm respectively, at optimal ratio 4:4:2).

To summarize, during the oil phase optimization it was found that Tocopheryl acetate (TA) and Isostearyl isostearate (ISIS) were compatible with all ROs; however, the oil phase components ratio must be adjusted to match the preferred RO. Therefore, it was not possible to overcome the initial differences between ROs when combining them with TA and ISIS. Interestingly, LE-NEs loaded with RO2 (the organic, unrefined, cold-pressed oil) had smaller particle sizes compared to other ROs, regardless of RO2 being used as a single component or in combination with oil phase additives.

#### Water phase optimization − influence of glycerol and antioxidant hydro-glycolic extracts on LE-NE properties

Having in mind that modern dermo-cosmetic formulations are complex mixtures of oil and water-soluble active ingredients, surfactants, water, and in some cases additives (e.g. preservatives, rheological modifiers, fragrances), the same complexity is expected from nanoformulations. However, there is a lack of published research papers regarding the effect of polyols and hydro-glycolic plant extracts suitable for (dermo)cosmetic applications on PIC LE-NE formation and stability, especially in combination with mixed natural oil phases.

It is known that polyols can interact with surfactant molecules at the oil-water interface to reduce surface tension, change surfactant curvature and phase behavior of SOW mixtures; consequently, polyols can promote the formation of low-energy nanoemulsions and microemulsions of various types [34,35]. For example, it is known that glycerol can dehydrate the hydrophilic head groups in P80 molecules, therefore change the optimal surfactant curvature necessary for LE-NE formation [34,35]. Polyols are often present in skincare formulations, because they act as humectants/moisturizing agents [35], which was the reason to use glycerol in this study. The hydro-glycolic extracts of the whole red raspberry fruit (RE) and French oak fruit (FE) were also used, aiming to strengthen the antioxidant activity and stability of LE-NEs with their bioactive ingredients [6–10].

The Z- average droplet sizes and PDI values of the LE-NEs prepared with mixed oil phases (all four ROs/ ISIS/ TA) and glycerol/fruit extracts (RE, FE) were measured 24 h and one month after preparation, and they varied among the LE-NEs prepared with different ROs and different oil/ water phase additives (from ~120 nm to ~170 nm and from 0.05 to 0.125, respectively, as presented in S1 Table. Translucent or transparent LE-NEs were not formed, regardless of the type of the additive and its concentration. This was not unexpected since it is known that much higher polyol concentrations (e.g. 30 wt%) and SOR values are needed to obtain transparent or translucent LE-NEs [26,28]. The optimal glycerol content was 5 to 10 wt% in the water phase (i.e. 4 to 8 wt% of LE-NE total weight) and the optimal concentration of hydro-glycolic extracts was 5 wt% relative to the water phase (4 wt% of LE-NE) which was in agreement with their recommended concentration for dermal application. It was found that the optimal polyol/ hydro-glycolic extract concentrations were strongly related to the formation of the gel-like transient phase, which was a necessary step in this process. This means that all ingredient ratios that would disturb the formation of the gel-like transient phase should be avoided (e.g. polyol concentration above 15 wt% or hydro-glycolic extracts above 10 wt%, relative to the water phase).

Taking into consideration all tested variations, the LE-NEs prepared with RO2 were considered optimal since they had the smallest particle sizes and narrow PDI values (~122 to 145 nm, PDI ≤ 0.1) and they exhibited good stability after one month storage at room temperature (S1 Table). Overall, the information regarding water and oil phase composition acquired in this preformulation study may serve as a useful guideline for formulators working on the development of nanocarriers with natural ingredients.

### Formulation study of red raspberry seed oil-loaded LE-NEs: Physicochemical properties, stability and *in vitro* antioxidant activity

During the preformulation study, the most prominent finding was that the choice of red raspberry seed oil type was the crucial factor in LE-NE formulation development. It has been shown that all other formulation components had to be specifically adjusted to match the chosen red raspberry oil, and that the process must proceed via the transient LC gel phase. In the second phase of this study, a combined approach to product characterization was employed, i.e. a mix of conventional methods (PTPD, electrical conductivity, DLS/LD measurements, microscopical analysis, and rheological measurements) and the methods new to this field (Raman spectroscopy and textural analysis).

#### Raman spectroscopy study of red raspberry seed oils (ROs) and corresponding LE-NEs

Raman spectroscopy was employed to gain further insight into fine differences among red raspberry seed oils, as well as the corresponding LE-NEs, and to detect the interactions between LE-NE components. Raman spectroscopy is an advanced technique that can be used to determine the fatty acid profile, i.e. degree of unsaturation in vegetable oils [19,20], and to detects their minor components, for example, carotenoids [36,37] and tocopherols [38,39] responsible for biological activity. Moreover, it was recently reported that Raman spectroscopy can be used to elaborate the structural changes when incorporating oils and curcumin [25] or proteins [26] into nano-droplets, as well as to study interactions among nanoemulsion components.

**Fig 3:**
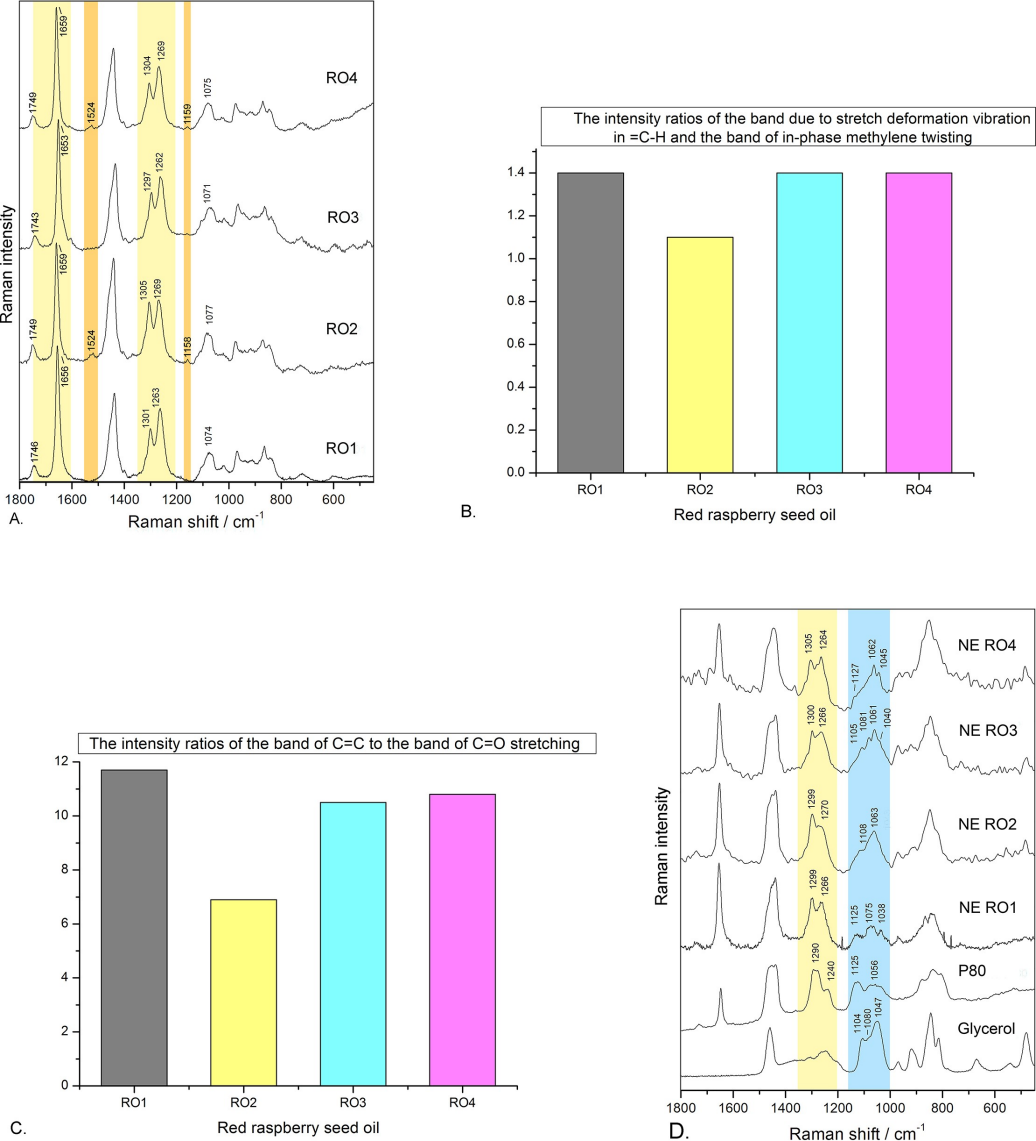
A. Raman spectra of different red raspberry seed oils (ROs): RO1 –cold-pressed, refined, non-organic oil; RO2 –cold-pressed, unrefined, organic oil; RO3 –CO_2_-extracted, unrefined, non-organic oil; RO4 –CO_2_-extracted, unrefined, organic oil. B. The intensity ratios of the bands due to stretch deformation vibration in = C-H and band of in-phase methylene twisting (I 1266/I 1305) in the Raman spectra of different red raspberry seed oils. C. The intensity ratios of the bands of C = C to C = O stretch (I1659/ I1749) in the Raman spectra of different red raspberry seed oils. D. Raman spectra of P80, glycerol and LE-NEs prepared with different ROs 48 h after preparation (LE-NE composition: P80 10 wt%, RO1/RO2/RO3/RO4 9 wt%, tocopheryl acetate 1 wt%, glycerol 8 wt%, water 72 wt%).

To the best of our knowledge, this is the first time the Raman spectra of different red raspberry seed oils are reported. Also, this study is the first to present the Raman investigation of the effect of small differences in raw materials (ROs) on the LE-NE formation and the interactions among the LE-NE components. As shown on Fig 3A, the fingerprint region of the Raman spectra of ROs (1800 to 800 cm^–1^) is dominated by two distinct spectral patterns: the pattern of the unsaturated fatty acids–UFAs (linoleic acid C18:2 ω6, α-linolenic acid C18:3 ω3 and oleic acid C18:1 ω9), and the pattern of the saturated fatty acids (palmitic C16:0 and stearic C18:0 acids). Therefore, the spectra of all four ROs were very similar, exhibiting the bands related to oils and fats positioned at: ~1747 cm^–1^ (C = O, ester carbonyl stretching), ~1659 cm^–1^ (C = C in fatty acid hydrocarbon chains), ~1440 cm^–1^ (scissoring deformation vibrations of CH2 groups in fatty acid chains), ~1305 cm^–1^ (in‐phase methylene twisting), ~1266 cm^–1^ (stretch due to deformation vibrations in = C–H), and ~1079 cm^–1^ (skeletal C‒C stretching vibrations) [19,20,36,37]. The most prominent observed difference refers to the relative intensity ratios of the bands related to the degree of unsaturation in the oil hydrocarbon chains: I1266/I1305 (Fig 3B) and I1659/I1749 (Fig 3C) ratios were noticeably higher in the case of RO1, RO3, and RO4 oils compared to RO2 oil. It was previously reported that the increase of unsaturation is observed as an increase in the intensity of the band at ~1266 cm^–1^ (C = C–H bending vibration band) [19,37] and the band at ~1659 cm^-1^ (C = C in fatty acid hydrocarbon chains) [20,36,37], where the band at ~1747 cm^–1^ (C = O, ester carbonyl stretching) can be used as an internal standard [20]. These spectral features were in accordance with the producers’ specifications (Table 1), where it was reported that RO2 had 6.62% of saturated fatty acids and 92.25% of unsaturated fatty acids while all other ROs had lower saturated acid content (3.09 to 4.04%) and higher content of total unsaturated acids (94.05 to 96.1%).

The second observed difference among the ROs Raman spectra was related to their carotenoid content which was not stated in the producers’ specifications. It was found that only the organic, unrefined oils–RO2 (cold-pressed) and RO4 (CO_2_-extracted) exhibited the bands assigned to β-carotene positioned at ~1524 cm^–1^ (C = C stretching attributed to carotenoids) and at ~1159 cm^–1^ (C−C stretching vibration mode) [36,37]. This was in accordance with the more intensive yellow to orange color of these organic oils (Table 1) and the reported findings that these bioactive ingredients can vary up to 2–4 times depending on the red raspberry variety [4] or the oil production technique [3,8]. Tocopherols were not detected in the ROs Raman spectra, which does not mean they are not present (e.g. producer specification included total tocopherol content for RO3 and RO4) because there is an overlap between the bands of fatty acids abundantly present in ROs and the bands of tocopherols [38]. A different experimental setup or sample preparation procedure (e.g. surface-enhanced Raman spectroscopy) would be necessary to elucidate their content [39]. Nevertheless, our focus remained on the influence of fatty acid profile on the LE-NE formation and properties, as these are the major constituents of ROs.

Regarding the Raman spectra of the LE-NEs prepared with various ROs while keeping all other components and production method identical, their peak characteristics are in line with the peaks of glycerol, P80, and ROs (Fig 3D), with some observed shifts of the characteristic bands. The ratio of the bands at ~1305 cm^–1^ and ~1266 cm^–1^ in the spectra of LE-NEs was changed compared to the observed trend in the spectra of ROs. The intensity of the band at ~1300 cm^–1^ was increased in all LE-NE spectra, and it can be correlated with the presence of the band at ~1290 cm^–1^ in spectrum of P80 (due to CH2 in-phase twist, and CH_2_ twist and rock vibrations in oleic acid) which contributes to its intensity in LE-NEs. Differences were also observed in the region at 1200–900 cm^−1^, which is the characteristic of hydrocarbon skeletal C–C stretching vibrations and stretching vibration of C–O bonds ν_s_(C–OH) (Fig 3D).

This could be assigned to the interactions between the LE-NE components (ROs, P80, glycerol, and water), causing shifts of the bands characteristic for pure P80 and glycerol (Fig 3D). In the spectra of pure glycerol, three distinctive bands were observed in this region at 1047, 1080 and 1104 cm^–1^. The bands at 1047 and 1104 cm^–1^ are assigned to ν_s_(C–OH) mode, while the band at 1080 cm^–1^ is ascribed to the twist (CH2) modes. Additionally, in the Raman spectrum of P80, two characteristic bands were observed at 1125 cm^-1^ ν_s_(C–OH) mode and a broad band at about 1056 cm^-1^, as the combination of ν_s_(C–OH) mode and twist (CH2) modes. It was also reported that in the Raman spectra of glycerol aqueous solution, a new ν_s_(C–OH) mode of less associated glycerol species (i.e. monomers and dimers) can appear at 1125 cm^–1^, because, upon dilution, higher-order glycerol oligomers would be expected to diminish at the expense of dimers and monomers [40]. The most important finding was that in the spectra of the RO2-loaded LE-NE, only two bands, at 1063 and 1108 cm^-1^, were observed. Since all pure ROs also have the band at ~ 1075 cm^-1^ (skeletal C‒C stretching vibrations), the new band at 1063 cm^-1^ in LE-NE spectra is probably the result of a new uniform structure formed from glycerol, P80, and RO2. This new structure was induced by the changed conformational order, packing and dynamical changes involving hydrocarbon chain and hydrogen bonding in the aqueous nanoemulsion environment. We propose that these interactions in the presence or RO2 have led to the formation of the most stable nanoemulsion with the smallest droplet sizes (RO2: 125.5 nm, RO1: 157.2 nm, RO3: 143.9 nm, RO4: 151.8 nm). It is important to note that in the LE-NEs prepared with other ROs, the band 1063 cm^-1^ was also present, but it was accompanied by the bands characteristic for pure P80 and glycerol, indicating different structural arrangements within the RO1, RO3 and RO4 nanoemulsions.

The results of the Raman spectroscopy study were in line with the screening/ preformulation phase where it was initially discovered that it was not possible to change one RO to another without a major impact on LE-NE structure and stability. Therefore, the use of Raman spectroscopy was critical in detecting the differences in ROs composition, to gain an insight into the interactions between the components of RO-loaded LE-NE. Based on the preformulation phase and the Raman study, the RO2-oil loaded LE-NEs were used for all further investigations.

#### Microscopical investigations–Optical (polarized) light microscopy, atomic force microscopy

**Fig 4:**
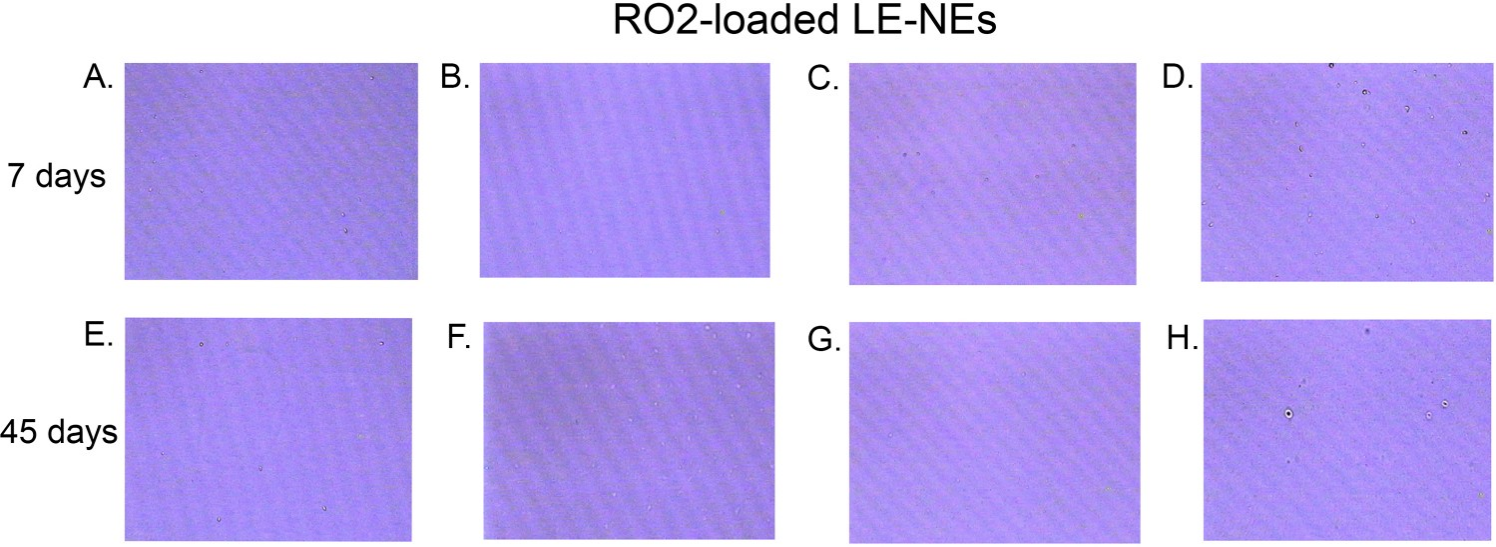
Optical microscopy study of LE-NEs prepared with RO2 –the cold-pressed, organic, unrefined seed oil: (A-D) after 7 days of storage at room temperature (RT); (E-H) after 45 days of storage at RT. All micrographs were taken at 400x magnification.

Microscopical investigations, as direct techniques complementary to indirect DLS/LD measurements [17,41] were employed during the PTPD study revealing that transient, semi-transparent, liquid-crystalline gel phases formed at SOR 1 (Fig 1, section 3) show lack of birefringence under polarized light. Since RO2 oil was found to be the most favorable for LE-NEs, the set of additional samples were prepared: the RO2-loaded gel phases with 30% water phase (G1−G4, Table 2) and the RO2-loaded LE-NEs with 80% water phase (F1−F4, Table 2). It was found that all investigated gel phases G1−G4 are isotropic regardless of the addition of ISIS (oil phase additive) and/or hydro-glycolic fruit extracts (RE, FE). The resulting RO2-loaded LE-NEs showed good stability after 45 days of storage at room temperature (Table 4). The micrographs presented in Fig 4 showed no visible signs of large aggregates in the LE-NEs after 7 and 45 days of storage at room temperature, which was in accordance with the narrow particle size distribution bellow 150 nm obtained via DLS and LD measurements (Table 4).

**Table 4:** Physicochemical stability of the selected RO2-loaded LE-Nes.

	Z-average (nm)		PDI		d10	d50	d90	pH		El. cond. (μS/cm)	
	24h	45d	24h	45d	45d			24h	45d	24h	45d
**F1**	131.0	130.3	0.079	0.070	0.110	0.142	0.183	6.603	6.650	71.433	61.167
st.dev.	1.877	3.252	0.008	0.027				0.031	0.010	0.251	0.352
**F2**	134.8	128.6	0.086	0.089	0.115	0.148	0.191	7.313	6.783	66.967	65.230
st.dev.	4.179	2.774	0.011	0.042				0.006	0.038	0.416	0.057
**F3**	122.3	123.1	0.054	0.093	0.108	0.140	0.179	6.647	6.423	94.567	79.367
st.dev.	3.427	2.706	0.033	0.012				0.006	0.015	0.115	0.153
**F4**	124.6	124.6	0.085	0.068	0.104	0.136	0.176	5.650	5.693	251.333	228.333
st.dev.	3.407	1.007	0.007	0.013				0.010	0.021	0.577	0.577

Z-average particle size, PDI, pH value and electrical conductivity 24 h after preparation, and after 45 days of storage at room temperature (RT). Parameters relevant to LD measurements (d10, d50, d90) were obtained after 45 days of storage at RT. All values represent means ± standard deviations of minimally three measurements, performed at RT.

**Fig 5:**
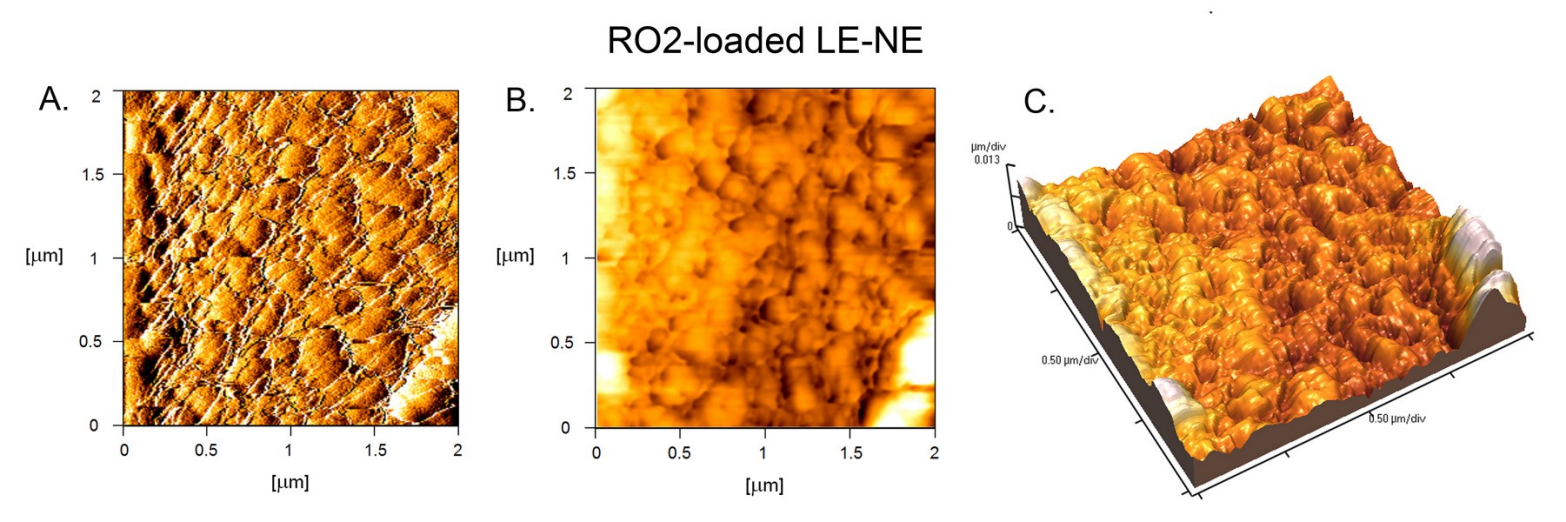
Atomic force microscopy of a representative stable LE-NE formulation (F1) prepared with RO2 –the cold-pressed, organic, unrefined red raspberry seed oil: (A) 2D error signal, (B) 2D topography, (C) 3D topography. The micrographs were taken 7 days after preparation.

AFM is known as a useful technique in the evaluation of the nanoemulsion internal structure [41] in the submicron range. As it can be seen from Fig 5, the RO2-loaded LE-NE (F1) consisted of “oval-shaped” nano-droplets, and the size range of these droplets was generally in line with DLS/LD findings. It was also observed that the oil droplets were closely packed, which was probably caused by the PIC production method and by the surfactant organization at the oil-water interface to achieve minimal energy [18].

#### Electrical conductivity measurements

**Fig 6:**
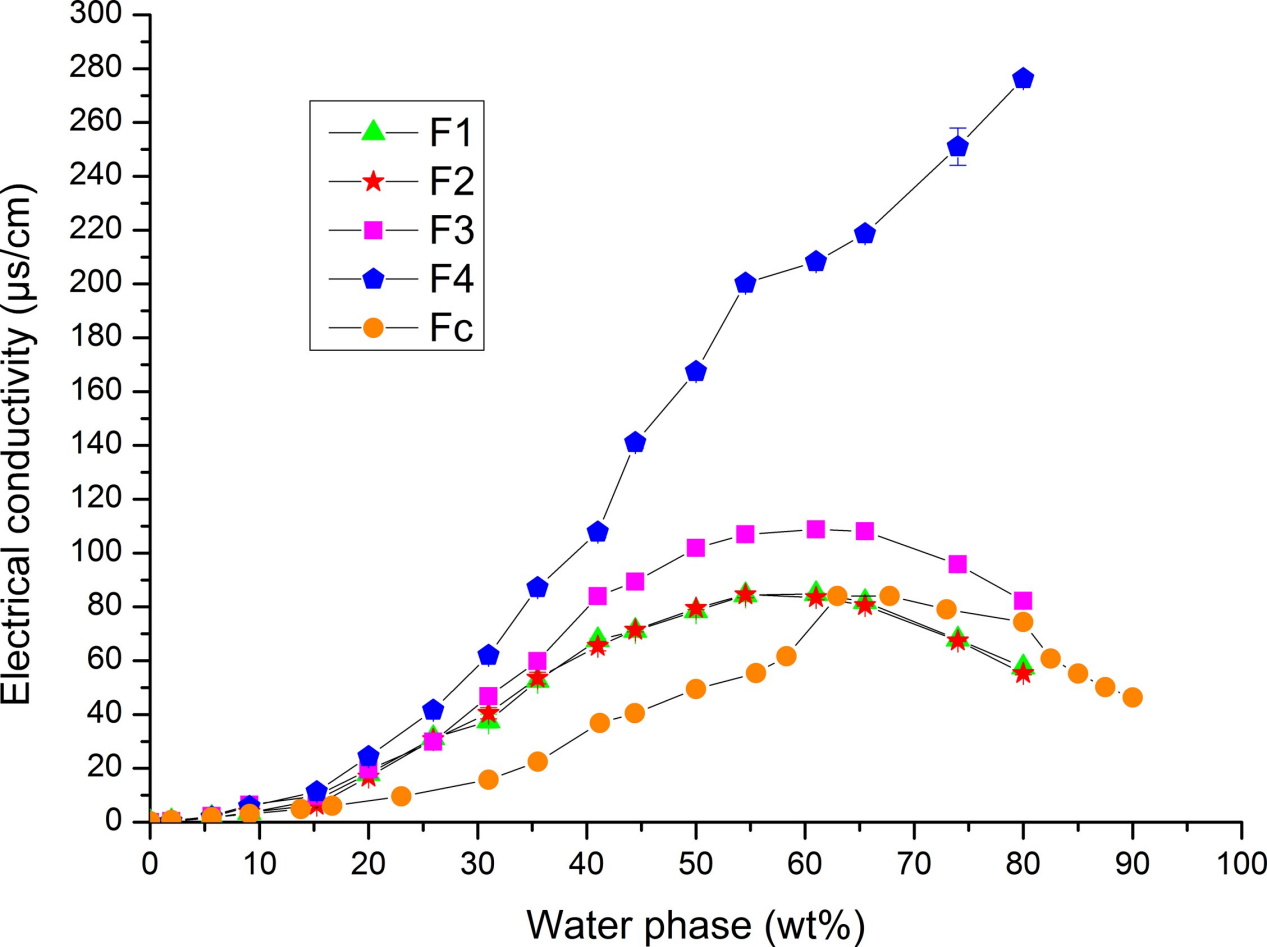
Electrical conductivity curves as a function of water phase content (wt%) along the PIC LE-NE formation pathway (of formulations F1−F4), or in a binary mixture (sample F control − P80/ 10 v/v% glycerol aqueous solution).

To gain further insight into the formation of stable RO2-loaded LE-NEs containing oil phase (ISIS), and/or water phase additives (RE, FE) via the PIC process (samples G1−G4, i.e. F1−F4, presented in Table 2), electrical conductivity measurements were carried out along the nanoemulsion formation pathway. As seen on Fig 6, the formation of LE-NE passed through several characteristic phases. At lower water content (up to about 20 wt% water phase) the values of electrical conductivity were very low, indicating that the water domains were separated by the outer oil phase, which corresponds to W/O emulsion system. When the water content increased to intermediate values (about 20 to 40 wt%), there was a sharp increase in electrical conductivity, indicating the existence of water channels (conductive pathways), corresponding to the bicontinuous cubic gel-like structure of high viscosity. When about 40 to 50 wt% water phase was present in the SOW system, the water gradually reached the outer phase and the system started to flow. Finally, at about 55 to 60 wt% water phase, the electrical conductivity reached its maximum values (for the formulations F1, F2, and F3) indicating that the water became the outer phase, which can be ascribed to the formation of liquid O/W LE-NE. With further increase of the water phase content (from 65 to 80 wt%), the electrical conductivity values decreased, which is a known phenomenon that can be explained by the dilution of the system [41].

The only LE-NE that exhibited elevated electrical conductivity upon dilution (up to the final 80 wt%) was F4. This behavior was caused by the specific composition of French oak fruit extract FE added to the LE-NE water phase. According to FE producer’s specifications [9] and recent publications with similar oak extracts [10,11] the FE extract (i.e. *Quercus petraea* fruit/acorn hydro-glycolic extract) represents a rich source of bioactives, e.g. tannins, flavonoids, proanthocyanidins, chlorophylls, phenolic acids. Therefore, the highest values of electrical conductivity are to be expected as the concentration of FE reaches its maximum value in LE-NE formulation. As for the other combination of ingredients, the variations in the oil phase (such as mixing RO2 with ISIS) or the addition of hydro-glycolic extract made from red raspberry fruit (RE) did not affect the electrical conductivity. Interestingly, the electrical conductivity values in the P80/ 10 v/v% glycerol solution (Fc–control sample) were much lower, and dense gel-like phases were absent during the dilution process.

In line with the results of a previous study [23], we conclude that the existence of cubic gel-like phases, as a necessary step in LE-NE formation, was induced by the specific composition of the oil phase and it was not jeopardized with the addition of RE and FE extract to the LE-NE water phase.

#### Rheological and textural analysis

*Oscillatory rheological measurements and textural analysis of gel-like phases*. Rheological and textural analysis were performed to gain a deeper insight into the structure of assumed cubic semi-solid transient LC phases, responsible for the LE-NE formation. In addition, these techniques were used to explore the potential link between several characteristic RO2-loaded gel-like phases containing 30 wt% water phase (samples G1–G4, Table 2) and the corresponding LE-NEs with different composition (the presence of oil phase additive–ISIS and/or hydro-glycolic extracts–RE, FE).

It is known that cubic phases are highly elastic LC isotropic phases [24,42,43] composed of surfactant micelles arranged in a three-dimensional lattice, which has been previously shown using oscillatory rheology coupled with textural analysis [24,35,44]. In a study similar to this one [24], using P80/Oil/Water systems with different types of oils, it was found that the micellar cubic phases occurred only in the presence of oil, which was in line with our observations during the PTPD study (Fig 1, section 3) and electrical conductivity measurements (Fig 6).

The most prominent characteristic of the cubic gel-phase is its "hard-gel" structure, a typical feature of cubic lattice [24,42,43]. Indeed, in this study the semi-transparent RO2-loaded gel-like phases G1−G4 did not flow when the glass vial was turned upside down, and no phase separation was observed seven days after preparation, indicating their capacity to incorporate a high amount of oil. Continuous flow tests showed that the Weissenberg effect occurred in all tested samples. Therefore, oscillatory rheological measurements were performed to assess their viscoelastic behavior, using parameters G'- elastic/storage modulus, G''- viscous/loss modulus and η*- complex viscosity.

**Fig 7:**
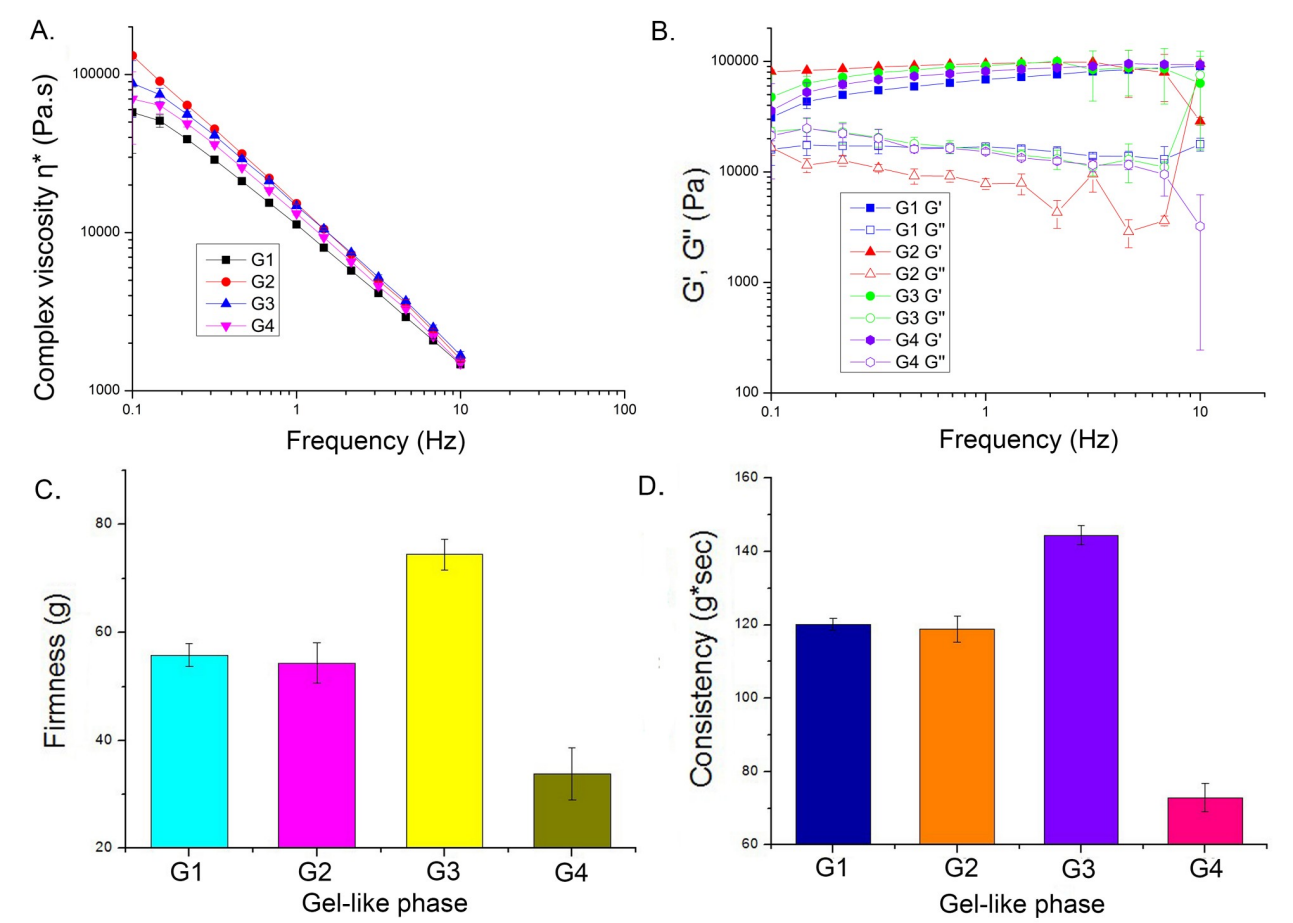
Oscillatory rheological measurements of the gel-like phases: (A) complex viscosity as a function of frequency; (B) G'–elastic modulus (filled symbols) and G''− viscous modulus (empty symbols) as a function of frequency; Textural analysis of the gel-like phases: (C) Firmness (g), (D) Consistency (g*sec).

As seen on Fig 7B, in all tested samples the values of elastic modulus (G') were four orders of magnitude higher than for the viscous modulus (G''), which indicates a solid or gel-like structure of the cubic lattice [42,43]. For all samples, the measured values of complex viscosity (η*) decreased as the frequency increased, without significant differences between the samples (p< 0.05) (Fig 7A). Despite some fine differences in the viscoelastic behavior of the tested LC phases, the overall trend for the elastic G' and viscous G'' moduli and η* as a function of frequency were the same for all tested samples (G1−G4). This finding indicates that all samples have similar, cubic gel-like structure, which was in accordance with the lack of birefringence.

Furthermore, textural analysis was performed to determine the mechanical properties of these gel-like phases (G1−G4), as a relatively novel approach to investigate the influence of the compositional changes on the transient gel structures [24,44]. Two parameters were analyzed: firmness − the maximum force required to attain a given deformation, and consistency–the work necessary to overcome the internal bonds within a sample in order to allow the immersion of the probe [45,46]. The results presented in Fig 7C and 7D showed that gel-like phases G1 and G2 had very similar values of firmness and consistency (a non-significant difference) regardless of the presence of ISIS in the G2 sample. The presence of antioxidant extracts in the gel-like phases (G3 and G4) influenced the gel structure in two opposite ways: RE extract caused an increase (G3), while FE caused a decrease of firmness and consistency (G4). It was previously reported that polyphenol-rich plant extracts can decrease the viscosity of emulsions, most likely due to interactions with surfactant molecules [47]. An additional explanation for the difference in G3 and G4 gel-like phase behavior could be found in the presence of different polyols in these hydro-glycolic extracts (propylene glycol in RE/ G3 and glycerol in FE/ G4, usually about 25 to 50 wt% of extract). Having in mind that many other bioactive molecules are present in these extracts (sugars, vitamins, fruit acids, phytopigments), several other possible interactions could arise among the components of the gel-like phase. It has been shown that plant extracts can influence the packing of liquid crystalline carriers, for example by inducing the change from hexagonal or lamellar to cubic [24]. However, when using the medium concentration of extracts (in this study, 5 wt% RE/FE in water) it was concluded that all tested gel-like phases had the cubic structure, leading to the formation of stable RO2-loaded nanoemulsions.

*Continuous rheological measurements of LE-NEs*. Shear rate flow tests were performed in order to gain further insight into the structure and characteristics of the O/W LE-NE formulations containing 80 wt% water phase (RO2-loaded LE-NEs samples F1−F4), such as the ease of application on the skin, as well as to evaluate the differences among them related to their composition (Table 2).

The continuous shear flow test showed that all samples (F1−F4) exhibited non-Newtonian shear-thinning flow behavior. LE-NE flow curves (S2 Fig, Supporting information) were fitted according to the power law: $\tau =K\cdot {\left(\dot{\gamma }\right)}^{n}$ where τ represents shear stress, $\dot{\gamma }$− shear rate, K − consistency index and n − flow index (n).

Consistency index and flow index were analyzed to assess the LE-NEs flow behavior, with similar values obtained 24 h and 7 days after preparation (Table 5). The flow indexes of all investigated LE-NEs were smaller than 1, which is assigned to the pseudo-plastic flow behavior. At the same time, K-indexes were very low, indicating low consistency of the samples. These features are considered desirable for topical pharmaceutical/cosmetic applications, because those emulsions are liquid, easy to apply to the skin surface, but remain on the applied site [44]. These fluid LE-NEs could be especially suitable for spray products or it could be packed in pipette bottles (e.g. face serums, hair care, and sun protection products).

**Table 5:** Flow characteristics of the selected RO2-loaded low-energy nanoemulsions: K-index and n-index 7 days after preparation.

Nanoemulsion	K-index	n-index
**F1**	0.0144 ± 0.0007	0.7417 ± 0.0107
**F2**	0.0149 ± 0.0004	0.7418 ±0.0063
**F3**	0.0114 ± 0.0003	0.7589 ± 0.0049
**F4**	0.0117 ± 0.0005	0.7589 ±0.0122

All values represent means ± standard deviations of minimally three measurements, performed at room temperature.

The statistical analysis of K- and n-indexes revealed that the samples with the same water phase (F1 and F2), but different oil phases did not differ significantly. The same was true for the samples with the same oil phase (F3 and F4) and different antioxidant extracts in the water phase, in which case the LE-NEs were more fluid. This is in accordance with previously reported findings that polyphenol-rich plant extracts can cause a decrease in viscosity, or they can influence other emulsion features such as particle sizes and stability [47]. Similar behavior was observed in our study, finding that samples F3 and F4 with RE/FE fruit extracts in the water phase had smaller particle sizes than the ones prepared with glycerol (F1, F2) in the water phase (Table 4). To conclude, in the case of LE-NEs with 80 wt% water phase, the flow behavior is dominantly affected by the composition of the water phase. However, in the case of the transient gel-like phases (G1−G4) with 30 wt% water phase, more complex interactions of oil and water phase components were observed.

#### *In vitro* antioxidant activity and storage stability

*In vitro antioxidant activity*. In order to compare the antioxidant activity of lipophilic (red raspberry seed oil–RO2) and hydrophilic antioxidants (red raspberry–RE and French oak–FE fruit extracts), the *in vitro* method of assessing free radical scavenging before and after emulsification was used. Since formulated LE-NEs represent O/W systems, it was important to assess their ability to directly scavenge the ABTS^+^ radical (with nanoemulsions intact, in aqueous medium) which would result in the discoloration of the blue-green ABTS^+^ radical solution [7]. In the DPPH assay, methanol was used to dissolve tested nanoemulsions, assuming that the oil phase was extracted from the nano-droplets [25,41], and the discoloration of the purple DPPH radical solution is proportional to the antioxidant activity of the samples [25,41,48].

Table 6 presents the results of the screening study of the antioxidant activity of the raw materials and nanoemulsions F1 –F4 (composition list presented in Table 2) depending on the sample concentration. It should be noted that the highest possible dilution of nanoemulsion samples in the reaction mixture was 1:100 (100 μl/ 10 ml) for both assays since the LE-NEs were milky white. Therefore, the solution of relevant concentration of raw materials was chosen to reflect their wt% content in the LE-NEs.

**Table 6:** Antioxidant activity of low-energy nanoemulsions and raw materials used in this study: ABTS assay in PCS buffer, and DPPH assay in methanol.

Raw materials (μl/10 ml)	DPPH %INH	ABTS %INH	Nanoemulsions (μl/10 ml)	DPPH %INH	ABTS %INH
**RO2**			**F1**		
6*	6.43 ± 0.36	1.35 ± 0.14	50	6.77 ± 0.13	5.51 ± 0.17
12**	11.07 ± 0.18	2.28 ± 0.23	75	7.72 ± 0.26	9.68 ± 0.35
100	74.72 ± 0.26	20.95 ± 0.51	100	11.56 ± 0.31	11.47 ± 0.59
**FE**			**F2**		
1.7	82.02 ± 0.31	95.02 ± 0.99	50	3.93 ± 0.09	2.11 ± 0.01
4***	90.70 ± 0.23	95.75 ± 0.11	75	8.89 ± 0.02	2.95 ± 0.56
9	90.44 ± 0.11	96.34 ± 0.16	100	9.3 ± 0.11	2.65 ± 0.38
100	84.87 ± 0.04	96.84 ± 0.33			
**RE**			**F3**		
4****	2.01 ± 0.04	/	50	8.56 ± 0.22	6.16 ± 0.69
9	2.09 ± 0.06	/	75	10.76 ± 0.15	6.37 ± 0.27
100	7.13 ± 0.14	1.61 ± 0.034	100	12.96 ± 0.27	12.09 ± 0.12
			**F4**		
**Nanoemulsion control samples**			50	62.46 ± 0.36	75.99 ± 0.69
**F negative cont.**	1–2.5% INH. in both assays		75	78.17 ± 0.37	93.29 ± 0.79
**F positive cont.**	3–5% INH. in both assays		100	91.55 ± 0.31	92.33 ± 0.44

*corresponds to the same wt% of raw material in 100 μl/10ml of F2 nanoemulsion

**corresponds to the same wt% of raw material in 100 μl/10ml of F1, F3 and F4 nanoemulsions

***corresponds to the same wt% of raw material in 100 μl/10ml of F4 nanoemulsion

****corresponds to the same wt% of raw material in 100 μl/10ml of F3 nanoemulsion

F negative control: Isostearyl isostearate—10 wt% in the nanoemulsion oil phase, without RO2, or any other antioxidants

F positive control: Tocopheryl acetate—1%, Isostearyl isostearate - 9wt% in the nanoemulsion oil phase, without RO2 or any other antioxidants

/ Antioxidant activity was not detected

The results are expressed as the Percentage of Inhibition (%INH) of free radical

Our findings confirmed the concentration-dependent antioxidant activity of pure RO2 in both mediums: in the ABTS assay, the percentage of inhibition (%INH) was 20.95, while in DPPH it was much higher (74.72%). The RO2 free radical scavenging effect in the DPPH test is mainly attributed to its lipophilic antioxidant molecules (carotenoids and tocopherols) [1–4] while the RO2 activity confirmed by the ABTS test in an aqueous medium can be attributed to the presence of hydrophilic antioxidants in raspberry oil [8]. In the DPPH assay, the oil was dissolved in methanol, hence the measured activity of RO was much higher. The significant finding was that FE exhibits very high antioxidant activity in both assays (> 82%) even in the minimal tested concentration (1.67 μl/ 10 ml). This was in accordance with the previous study that revealed the high antioxidant performance of oak extracts, especially the ones made from fruit (acorn) [10]. On the contrary, RE did not show antioxidant activity in the ABTS assay, while minimal antioxidant activity was observed in the DPPH test (7.13%) at the highest tested concentration.

Regarding the potential application on the skin, the results obtained for the LE-NEs were of particular interest: in DPPH assay the concentration dependence of antioxidant activity was confirmed, while for the ABTS test it was not clearly manifested. Overall, the antioxidant activity of nanoemulsions was consistent with the antioxidant activity of the raw materials, taking into account their wt% in LE-NEs.

At the highest tested LE-NEs concentration, for all samples the obtained results showed a similar trend of antioxidant activity using both assays (specifically good correlation was observed for samples F1, F3, and F4, Table 6). The exception was F2 –the LE-NE sample with 4.5 wt% RO2 in the oil phase since it showed negligible %INH in the ABTS assay, but in the DPPH assay, when the oil is extracted from the LE-NE, the result was higher (2.65% vs. 9.30%). In both assays, F3 and F4 had higher antioxidant activity than F1 (all three samples have 9 wt% RO2) indicating that hydro-glycolic extracts RE and FE contributed to overall antioxidant activity. While for F3 the antioxidant activity was only slightly improved, for F4 it was very high (92.33% ABTS, 91.55% DPPH). This can be attributed to the presence of 5 wt% FE in the F4 water phase based on the finding that the French oak extract itself has a very high capacity to scavenge both free radicals used in this study.

*Storage stability*. The low-energy nanoemulsion formulations loaded with RO2 (F1−F4) showed good stability at room temperature 45 days of storage (Table 4), with no major changes detected in mean droplet sizes, PDI, pH values and electrical conductivity. This was consistent with the lack of visible aggregates up to 45 days of storage (Fig 4). However, red raspberry seed oil is known to be prone to rapid oxidation because of the high content of unsaturated fatty acids [1,28]. Like other natural oils, RO can be prone to hydrolysis, where free fatty acids are released from fatty acid esters, and the pH value is decreased [49]. It is also known that P80-based LE-NEs can be very unstable at high temperatures, due to the changes in surfactant HLB value [28].

During the preliminary 45 days long stability study of formulations F1-F4 conducted at 4, 25 and 40°C (presented in the Supporting information section) it was found that French oak exhibited a remarkable stabilizing effect in nanoemulsion F4 at 40°C. Unexpectedly, Tocopheryl acetate was effective only at lower temperatures (4, 25°C) in all tested formulations. The stabilizing effect of FE finding is in line with the known positive effects of polyphenol-rich plant extracts in topical formulations, including high antioxidant activity, improved stability due to their positioning at the surfactant-oil interface and positive influence on emulsion viscosity [47]. Therefore, French oak fruit extract can be used as an effective ingredient to boost antioxidant activity and to protect low-energy nanoemulsions containing thermo-sensitive natural ingredients, such as red raspberry seed oil.

#### *In vitro* biological activity

In order to gain insight into the safety profile of the formulated nanoemulsions with antioxidant natural extracts used in this study, an *in vitro* test on normal lung fibroblasts (MRC-5 cells) was performed. In addition, the potential biological effects against tumor cells (i.e. human melanoma–Fem-X and human cervical adenocarcinoma–HeLa) were also investigated, based on the standard MTT test procedure [50] and some previous research related to the topical application of natural compounds in nanoformulations [51]. There is a lack of comprehensive information in the literature regarding these particular cell lines, but it was confirmed that various berry extracts inhibit breast and gastric cancer cells at 250 μg/ml [52]. The IC50 value of 103 μg/ml was reported in a study performed on colon adenocarcinoma [53].

**Fig 8:**
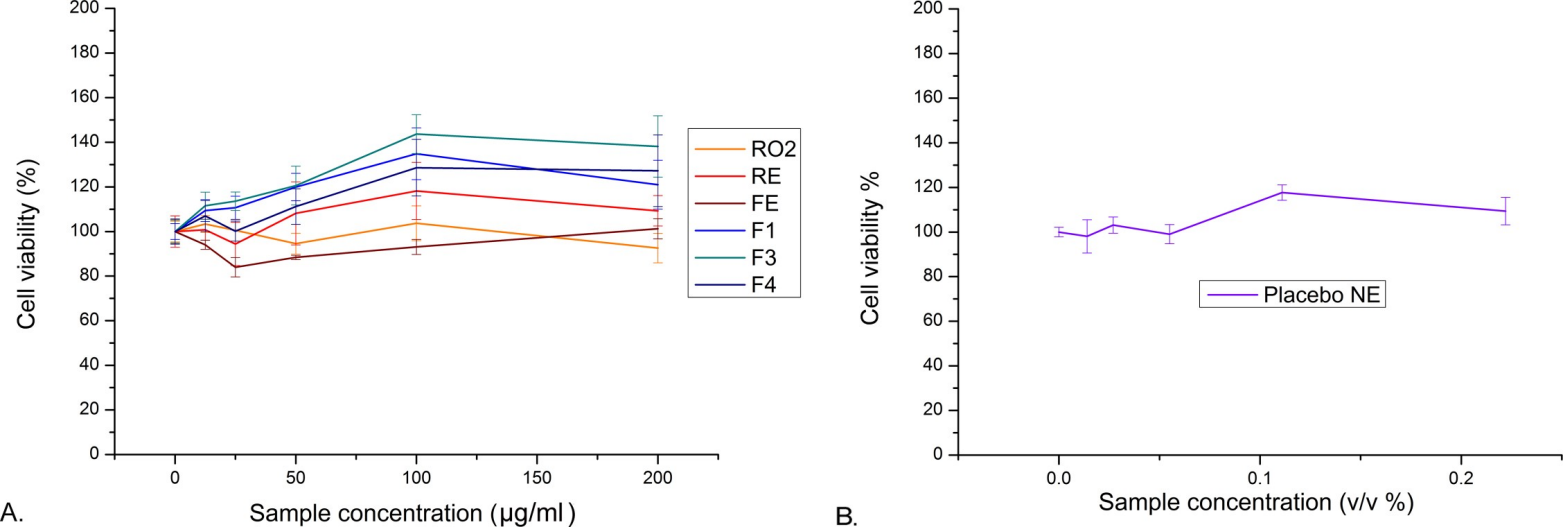
Safety assessment by MTT test: (A) Effect of the raw materials and the LE-NE formulations on MRC-5 cell line, (B) Effect of the placebo NE on MRC-5 cells. Obtained data show cell viability % depending on the sample concentration. Each experiment was repeated three times and the results were presented as the mean value ± SD.

**Fig 9:**
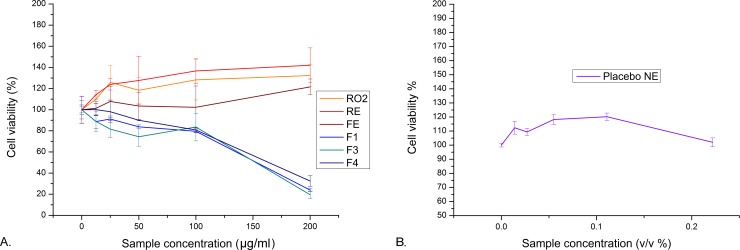
Anti-proliferative effect on HeLa cells by MTT test: (A) Effect of the raw materials and the LE-NE formulations on HeLa cell line, (B) Effect of the placebo NE on HeLa cells. Obtained data show cell viability % depending on the sample concentration. Each experiment was repeated three times and the results were presented as the mean value ± SD.

As seen on Fig 8A, normal fibroblasts were unaffected by the raw materials alone (RO2, RE and FE) or in the form of nanoemulsions (F1, F3 and F4; Tables 2 and 6). The safety of placebo NE was confirmed in Fig 8B (IC50 > 200 μg/ml). Fem-X cells were also non-sensitive to the tested raw materials and respective nanoemulsions, with IC50 > 200 μg/ml (S3A and S3B Fig, Supporting information). Interestingly, the anti-proliferative effect was pronounced on HeLa cells, but only in the presence of LE-NEs (Fig 9A and 9B, Table 7).

**Table 7:** MTT assay results: Concentrations of red raspberry seed oil or red raspberry seed oil with the addition of RE/ FE extract applied in the form of nanoemulsions that induced 50% decrease in the cell survival (IC50).

Sample	MRC-5	HeLa
F1	>200	153.12 ± 25.66
F3	>200	152.41 ± 14.62
F4	>200	163.72 ± 10.52

All data are results of the three independent experiments, each carried out in triplicate and the results are expressed in μg/ml

These results confirm the notion that raw materials’ activity can be improved in nanosized formulations, which has been the main drive to produce such formulations [48, 50]. The obtained IC50 values on HeLa cells were 153.12 ± 25.66, 152.41 ± 14.62 and 163.72 ± 10.52 μg/ml, for LE-NE formulations F1, F3 and F4, respectively (Table 7). Since there were no significant differences between IC50 values of sample F1 with RO2 in the oil phase and RO2-loaded samples prepared with the additional hydrophilic antioxidants RE (F3) and FE (F4), it can be concluded that RO2 is the main ingredient responsible for the observed effects on HeLa cells. The discrepancy between *in vitro* free radical scavenging effect and anti-proliferative effects on human cells (such as in the case of samples FE and F4), was previously reported in the literature [54] and it clearly indicates the necessity for comparative studies in the future, to reveal the precise mechanism of action.

## Conclusions

In this study a combined approach in formulation development and optimization of low-energy nanoemulsions was employed, with particular contribution made through the PTPD study coupled with Raman investigations in detecting the type of red raspberry seed oil as the key formulation parameter. Out of the four oil variations studied, the cold-pressed, unrefined, organic grade oil (RO2) was the most suitable one. In addition, the textural analysis was essential in confirming that cubic gel-like transient phase was a necessary step in the preparation of RO2-loaded LE-NEs via PIC method, and that all ingredients that disturb this step are unfavorable (e.g. polyol concentration above 15 wt% or fruit hydro-glycolic antioxidant extracts above 10 wt%, relative to the water phase). The synergistic free radical scavenging effect was pronounced in LE-NEs with combined lipophilic (in RO2) and hydrophilic antioxidants (in FE) with very high DPPH and ABTS results, while also insuring good stability at 40°C. All raw materials and LE-NEs showed satisfactory safety profiles in the MTT test on MRC-5 cells. Importantly, the anti-proliferative effect was more pronounced on HeLa cells when using nanoemulsions than neat ingredients, confirming the notion that bioactivity or raw materials can be improved by using appropriate nanocarriers.

The results of thorough structural and physico-chemical investigations completed in this study have established a number of individual and interactive effects of natural raw materials on the low energy nanoemulsion formation, some of them explored for the first time. The ultimate purpose of this work was to offer theoretical and practical insights that would enable the formulation of stable, safe and efficacious nanoemulsion carriers with natural actives for topical application.

## Supporting information

S1 File The following information are available: Experimental setup for Raman spectroscopy and Atomic force microscopy analysis; Antioxidant assays (ABTS and DPPH) and Storage stability study of nanoemulsions F1 – F4 (composition presented in Table 2). (DOCX)

S1 Table Preformulation study of different red raspberry seed oils (ROs): Z-average droplet size (nm) and PDI of nanoemulsions prepared with mixed oil (ROs/Tocopheryl acetate-TA/Isostearyl isostearate-ISIS) and mixed water phases with added glycerol (GLY), or antioxidant fruit extracts of red raspberry− RE/ French oak− FE. (DOCX)

S1 Fig Interactions between the red raspberry seed oil of different type (RO type) and SOR: A. Z-average droplet size as a function of SOR, B. Mean polydispersity index as a function of SOR.
(TIF)

S2 Fig Representative flow curve of red raspberry seed oil-loaded low-energy nanoemulsion F1. (TIF)

S3 Fig Cell viability assay performed on Fem-X human malignant melanoma cells: (A) Effect of the raw materials and the LE-NE formulations, (B) Effect of the placebo NE. Obtained data represent cell viability % of the cell culture depending on the sample concentration. Each experiment was repeated three times and the results were presented as the mean value ± SD.
(TIF)

S4 Fig Optical density at 570 nm of the MRC-5 normal human lung fibroblast cells: (A) Effect of the raw materials and the LE-NE formulations, B) Effect of the placebo NE.
(TIF)

S5 Fig Optical density at 570 nm of the HeLa human adenocarcinoma cells: (A) Effect of the raw materials and the LE-NE formulations, B) Effect of the placebo NE.
(TIF)

S6 Fig Optical density at 570 nm of the Fem-X human malignant melanoma cells: (A) Effect of the raw materials and the LE-NE formulations, B) Effect of the placebo NE. Obtained data represent optical density of the cell culture depending on the sample concentration. Each experiment was repeated three times and the results were presented as the mean value ± SD.
(TIF)
