# Supplementary material for: Low-energy nanoemulsions as carriers for red raspberry seed oil: Formulation approach based on Raman spectroscopy and textural analysis, physicochemical properties, stability and in vitro antioxidant/ biological activity
Source: PLoS One. 2020 Apr 16;15(4):e0230993. doi: 10.1371/journal.pone.0230993 (PMC7161953; doi:10.1371/journal.pone.0230993)
Supplement: S1 File — (DOCX) [file pone.0230993.s001.docx]

# Supporting information for

**Low energy nanoemulsions as carriers for red raspberry seed oil: formulation approach based on Raman spectroscopy and textural analysis, physicochemical properties, stability and *in vitro* antioxidant/ biological activity**

# **Materials and methods**

## **Atomic force microscopy**

Before the analysis, LE-NE samples were diluted with ultra-pure water (1:500 v/v), and 10 µL of diluted sample was placed on circular mica substrate (Highest Grade V1 AFM Mica Discs, Ted Pella Inc., Redding, California, USA) and dried in vacuum. Morphology of the samples was studied by the atomic force microscope with AutoProbe CP-Research SPM (TM Microscopes-Bruker) using a 90 µm large area scanner. Two types of images, topographical image, and “error signal” were taken and analyzed using the software Image Processing and Data Analysis Version 2.1.15 and SPMLab Analysis, DI SPMLab NT Ver. 6.0.2. Because of the nature of the samples, the noncontact mode was applied. AFM measurements were performed in air using noncontact probes Bruker phosphorous (n)-doped silicon Tap300, model MPP-11123-10 with aluminum reflective coating and symmetric tip. During measurements, the cantilever driving frequency was 300 kHz. Two types of images, topographical image, and “error signal” were taken and analyzed using the software Image Processing and Data Analysis Version 2.1.15 and SPMLab Analysis, DI SPMLab NT Ver. 6.0.2.

## **Raman spectroscopy**

Different samples were investigated: red raspberry seed oils (ROs), glycerol, Polysorbate 80 and nanoemulsions that were prepared in the same manner with identical composition, except for the type of RO. Recording conditions were controlled to the fullest extent possible. All spectra were recorded the same day, after instrument alignment and calibration, and under the same conditions: 2.1-μm laser-focused spot size on the surface of the sample through a 10 × microscope objective; power of the laser (780 nm) was kept constant at 10 mW, exposure time was identical for all measurements, as well as other variable instrumental parameters. Also, the use of gold plates with well-defined sample wells (EZ-Spot Micro Mounts support from Thermo Scientific), which always contained the same sample volume (5 µL), eliminated the influence of variations in the laser focus on the samples between individual measurements. The effect of the potential inhomogeneity of the samples on their spectra was eliminated by multiple repetitions of the spectra recorded for each sample. For each sample, the average Raman spectrum was calculated, using Thermo Scientific OMNIC software, from the five measurements recorded from 2000 cm^−1^ to 300 cm^−1^ by taking a new aliquot (5 µL) of the samples each time at room temperature. The spectra were recorded using an exposure time of 10 seconds and 10 exposures per spectrum.

## ***In vitro* antioxidant activity**

### **ABTS assay**

Firstly, the ABTS radical cation (ABTS^+^) was prepared in the reaction of 10 mL ABTS (7 mM) in PCS buffer with 176 µL of 140 mM potassium persulphate solution (2.45 mM final concentration). The mixture was kept for 16 h in a dark glass volumetric flask, at room temperature. After that, the ABTS^+^ solution was diluted with PCS buffer to obtain the absorbance of 0.7±0.05 units (measured at 734 nm using a spectrophotometer). Trolox solutions of different concentrations (2.5 mM, 1.25, 0.625, 0.3125 and 0.15625 mM) were prepared in PCS buffer and used as standards. Free radical scavenging was tested by mixing various concentrations of raw materials (1.7 ̶ 100 µL of raspberry seed oil or antioxidant fruit extracts) or nanoemulsions (50 – 100 µL) with ABTS^+^ solution up to 10 ml. The mixtures were kept for 6 minutes at room temperature (with slight shaking repeated every minute), and the decrease in absorbance was measured at 734 nm.

It should be noted that 2 different blanks were used (3000 µL pure PCS buffer for extracts, and for LE-NEs 30 µL in 2970 µL PCS buffer) because LE-NEs were not transparent at test dilution ratios. Therefore, it was necessary to annul the absorbance of LE-NE solutions at 734 nm.

### **DPPH assay**

For DPPH assay 100 ml of 0.004% DPPH radical (DPPH) solution in methanol (test solution) and 100 mg/L of Trolox solution (standard) in methanol was prepared. The free radical scavenging activity was tested by mixing 1.7 ̶ 100 µL of each sample (raw material or nanoemulsion) with 5 ml DPPH test solution, and methanol was added up to 10 ml. Trolox solutions of different concentrations (0.5, 1, 1.5, 2, 2.5 and 3 mg/L) were prepared by mixing the needed amounts of Trolox standard solution with a 5 ml DPPH test solution, and methanol was added up to 10 ml. The samples or standards were kept in dark glass volumetric flasks, with slight shaking every 5 minutes, at room temperature. After 30 minutes, the absorbance was measured at 514 nm with a spectrophotometer (Beckman DU 650). The blank for all samples and Trolox was pure methanol.

## **Storage stability study**

Storage stability assessment (45 days stability study at 4, 25 and 40°C) of the selected RO2-loaded LE-NEs (see Table 2) was performed using pH, electrical conductivity and DLS measurements. The pH was measured with undiluted LE-NE samples using HI9321 microprocessor pH meter (Hanna Instruments Inc., Ann Arbor, Michigan, USA), whereas electrical conductivity was measured using SENSION+ EC71 conductivity meter (HACH, Loveland, Colorado, USA). All measurements were done in triplicate at room temperature, 24 to 48 hours after preparation and upon storage of samples at different temperatures.

# **Results and discussion**

## **S1 Table. Preformulation study of different red raspberry seed oils (ROs):** Z-average droplet size (nm) and PDI of nanoemulsions prepared with mixed oil (ROs/Tocopheryl acetate-TA/Isostearyl isostearate-ISIS) and mixed water phases with added glycerol (GLY), or antioxidant fruit extracts of red raspberry− RE/ French oak− FE. The values represent means of three repeated measurements, 24 hours and one month after preparation. Samples with signs of creaming or aggregation are marked as unstable (unst.)

| **WATER PHASE** | **GLY** | | | | **RE** | | | | **FE** | | | |
| --- | --- | --- | --- | --- | --- | --- | --- | --- | --- | --- | --- | --- |
|  | 4 wt% | | 8 wt% | | 4 wt% | | 8 wt% | | 4 wt% | | 8 wt% | |
| **OIL PHASE** | 24h | 1m | 24h | 1m | 24h | 1m | 24h | 1m | 24h | 1m | 24h | 1m |
| **RO1** 9wt%  TA 1wt% | 144.6 | 146.6 | 157.2 | 153.5 | 165.3 | 168.7 | 151.6 | 154.9 | 151.6 | 152.3 | 131.3 | 134.7 |
|  | 0.108 | 0.122 | 0.074 | 0.110 | 0.097 | 0.096 | 0.108 | 0.089 | 0.095 | 0.102 | 0.085 | 0.084 |
| **RO1** 4wt%  ISIS 4wt%  TA 2wt% | 148.3 | 146.3 | 140.2 | 139.7 | 149.5 | 154.5 | 153.3 | 159.57 | 145.3 | 146.2 | 140.4 | 140.8 |
|  | 0.081 | 0.098 | 0.060 | 0.129 | 0.099 | 0.084 | 0.105 | 0.092 | 0.092 | 0.06 | 0.116 | 0.125 |
| **RO2** 9wt%  TA 1wt% | 141.7 | 140.0 | 131.3 | 130.3 | 122.3 | 129.1 | 128.4 | 127.1 | 124.6 | 124.5 | 130.2 | 127.2 |
|  | 0.054 | 0.080 | 0.079 | 0.070 | 0.054 | 0.093 | 0.066 | 0.082 | 0.085 | 0.068 | 0.085 | 0.104 |
| **RO2**  4.5wt%  ISIS 4.5wt%  TA 1wt% | 129.6 | 125.6 | 134.8 | 128.6 | 134.1 | 131.7 | 140.2 | 142.3 | 124.8 | 125.8 | 139.7 | 145.4 |
|  | 0.051 | 0.066 | 0.086 | 0.089 | 0.090 | 0.084 | 0.095 | 0.092 | 0.093 | 0.100 | 0.077 | 0.100 |
| **RO3**  8wt%  TA 2wt% | 147.7 | 148.4 | 161.4 | 159.8 | 147.8 | 150.2 | 142.2 | 144.1 | 156.7 | 155.9 | 170.9 | 158.6 |
|  | 0.107 | 0.108 | 0.081 | 0.098 | 0.137 | 0.090 | 0.084 | 0.100 | 0.131 | 0.124 | 0.139 | 0.081 |
| **RO3** 4wt%  ISIS 4wt%  TA 2wt% | 144.9 | 144.6 | 156.6 | 170.6 | 134.9 | 141.2 | 169.3 | 171.8 | 143.7 | 144.3 | 155.9 | 157.4 |
|  | 0.111 | 0.101 | 0.089 | 0.134 | 0.126 | 0.099 | 0.103 | 0.125 | 0.093 | 0.127 | 0.103 | 0.133 |
| **RO4** 8wt%  TA 2wt% | 138.5 | 142.5 | 146.9 | 147.6 | 143.3 | 146.2 | 157.4 | 162.3 | 156.4 | unst. | 159.2 | unst. |
|  | 0.111 | 0.100 | 0.121 | 0.133 | 0.114 | 0.107 | 0.142 | 0.124 | 0.089 | unst. | 0.120 | unst. |
| **RO4**  4wt%  ISIS 4wt%  TA 2wt% | 152.9 | 154.3 | 135.5 | unst. | 135.4 | 135.0 | unst. | unst. | 130.1 | 136.4 | unst. | unst. |
|  | 0.112 | 0.104 | 0.106 | unst. | 0.103 | 0.089 | unst. | unst. | 0.135 | 0.106 | unst. | unst. |

## **Storage stability study**

Red raspberry seed oil (RO) is known to be prone to rapid oxidation because of the high content of unsaturated fatty acids [1,28]. Like other natural oils, RO can be prone to hydrolysis, where free fatty acids are released from fatty acid esters, and pH value is decreased [44]. It is also known that Polysorbate 80-based LE-NEs can be very unstable at high temperatures, due to the changes in surfactant HLB value [28]. To assess the preliminary stability of raspberry seed oil-loaded low energy nanoemulsions (LE-NEs), a 45 days stability study was conducted at 4°C (refrigeration conditions or storage in a cold climate), 25°C (ambient storage in a mild climate), and 40°C (ambient storage in a hot climate). Interestingly, at all temperatures, the Z-average sizes, and PDI values were roughly the same after 45 days of storage (up to 5% increase was observed) proving that Polysorbate 80-based nanocarriers are suitable for red raspberry seed oil and antioxidant hydro-glycolic extracts investigated in this study. The LE-NEs stored at 4°C were reasonably stable, with only moderate changes observed in pH value or electrical conductivity. The pH values increased in all LE-NEs (from initial values of 6.01, 6.02, 5.08 and 5.09 to 6.15, 6.23, 5.62 and 5.79 for F1, F2, F3, and F4, respectively). The electrical conductivity values also increased from initial values of 66.9, 63.23, 91.4 and 235.0 to 87.0, 78.0, 116.2 and 274.7 µS/cm for F1, F2, F3, and F4, respectively. As expected, the situation drastically changed at 40°C: the pH value decreased from the above-mentioned initial values to 3.57, 3.65, 3.84 and 4.7 for F1, F2, F3, and F4, respectively, whereas the electrical conductivity increased to 169.5, 166.2, 172.9 and 282.7 µS/cm for F1, F2, F3 and F4, respectively.

From the obtained results it can be concluded that the stability of red raspberry seed oil loaded LE-NEs was highly compromised at higher temperatures; however, the addition of antioxidant extracts in the NE water phase improved it significantly. RE extract exhibited mild protective effect, while FE extract ensured the satisfactory stability at 40°C, which was also confirmed with intensive rancid odor observed for F1 and F2 samples and a less intensive smell for F3. F4 was the only LE-NE formulation not exhibiting the distinctive rancid smell after 45 days of storage at 40°C, confirming the protective effect of FE extract. Therefore, it could be considered as the multifunctional additive of choice for the red raspberry LE-NEs intended for cosmetic and dermatological applications.
