# Supplementary material for: Low-energy nanoemulsions as carriers for red raspberry seed oil: Formulation approach based on Raman spectroscopy and textural analysis, physicochemical properties, stability and in vitro antioxidant/ biological activity
Source: PLoS One. 2020 Apr 16;15(4):e0230993. doi: 10.1371/journal.pone.0230993 (PMC7161953; doi:10.1371/journal.pone.0230993)
Supplement: S1 Table — (DOCX) [file pone.0230993.s002.docx]

## **S1 Table. Preformulation study of different red raspberry seed oils (ROs):** Z-average droplet size (nm) and PDI of nanoemulsions prepared with mixed oil (ROs/Tocopheryl acetate-TA/Isostearyl isostearate-ISIS) and mixed water phases with added glycerol (GLY), or antioxidant fruit extracts of red raspberry− RE/ French oak− FE. The values represent means of three repeated measurements, 24 hours and one month after preparation. Samples with signs of creaming or aggregation are marked as unstable (unst.)

| **WATER PHASE** | **GLY** | | | | **RE** | | | | **FE** | | | |
| --- | --- | --- | --- | --- | --- | --- | --- | --- | --- | --- | --- | --- |
|  | 4 wt% | | 8 wt% | | 4 wt% | | 8 wt% | | 4 wt% | | 8 wt% | |
| **OIL PHASE** | 24h | 1m | 24h | 1m | 24h | 1m | 24h | 1m | 24h | 1m | 24h | 1m |
| **RO1** 9wt%  TA 1wt% | 144.6 | 146.6 | 157.2 | 153.5 | 165.3 | 168.7 | 151.6 | 154.9 | 151.6 | 152.3 | 131.3 | 134.7 |
|  | 0.108 | 0.122 | 0.074 | 0.110 | 0.097 | 0.096 | 0.108 | 0.089 | 0.095 | 0.102 | 0.085 | 0.084 |
| **RO1** 4wt%  ISIS 4wt%  TA 2wt% | 148.3 | 146.3 | 140.2 | 139.7 | 149.5 | 154.5 | 153.3 | 159.57 | 145.3 | 146.2 | 140.4 | 140.8 |
|  | 0.081 | 0.098 | 0.060 | 0.129 | 0.099 | 0.084 | 0.105 | 0.092 | 0.092 | 0.06 | 0.116 | 0.125 |
| **RO2** 9wt%  TA 1wt% | 141.7 | 140.0 | 131.3 | 130.3 | 122.3 | 129.1 | 128.4 | 127.1 | 124.6 | 124.5 | 130.2 | 127.2 |
|  | 0.054 | 0.080 | 0.079 | 0.070 | 0.054 | 0.093 | 0.066 | 0.082 | 0.085 | 0.068 | 0.085 | 0.104 |
| **RO2**  4.5wt%  ISIS 4.5wt%  TA 1wt% | 129.6 | 125.6 | 134.8 | 128.6 | 134.1 | 131.7 | 140.2 | 142.3 | 124.8 | 125.8 | 139.7 | 145.4 |
|  | 0.051 | 0.066 | 0.086 | 0.089 | 0.090 | 0.084 | 0.095 | 0.092 | 0.093 | 0.100 | 0.077 | 0.100 |
| **RO3**  8wt%  TA 2wt% | 147.7 | 148.4 | 161.4 | 159.8 | 147.8 | 150.2 | 142.2 | 144.1 | 156.7 | 155.9 | 170.9 | 158.6 |
|  | 0.107 | 0.108 | 0.081 | 0.098 | 0.137 | 0.090 | 0.084 | 0.100 | 0.131 | 0.124 | 0.139 | 0.081 |
| **RO3** 4wt%  ISIS 4wt%  TA 2wt% | 144.9 | 144.6 | 156.6 | 170.6 | 134.9 | 141.2 | 169.3 | 171.8 | 143.7 | 144.3 | 155.9 | 157.4 |
|  | 0.111 | 0.101 | 0.089 | 0.134 | 0.126 | 0.099 | 0.103 | 0.125 | 0.093 | 0.127 | 0.103 | 0.133 |
| **RO4** 8wt%  TA 2wt% | 138.5 | 142.5 | 146.9 | 147.6 | 143.3 | 146.2 | 157.4 | 162.3 | 156.4 | unst. | 159.2 | unst. |
|  | 0.111 | 0.100 | 0.121 | 0.133 | 0.114 | 0.107 | 0.142 | 0.124 | 0.089 | unst. | 0.120 | unst. |
| **RO4**  4wt%  ISIS 4wt%  TA 2wt% | 152.9 | 154.3 | 135.5 | unst. | 135.4 | 135.0 | unst. | unst. | 130.1 | 136.4 | unst. | unst. |
|  | 0.112 | 0.104 | 0.106 | unst. | 0.103 | 0.089 | unst. | unst. | 0.135 | 0.106 | unst. | unst. |
