# Supplementary figures and images for: Low-energy nanoemulsions as carriers for red raspberry seed oil: Formulation approach based on Raman spectroscopy and textural analysis, physicochemical properties, stability and in vitro antioxidant/ biological activity
Source: PLoS One. 2020 Apr 16;15(4):e0230993. doi: 10.1371/journal.pone.0230993 (PMC7161953; doi:10.1371/journal.pone.0230993)

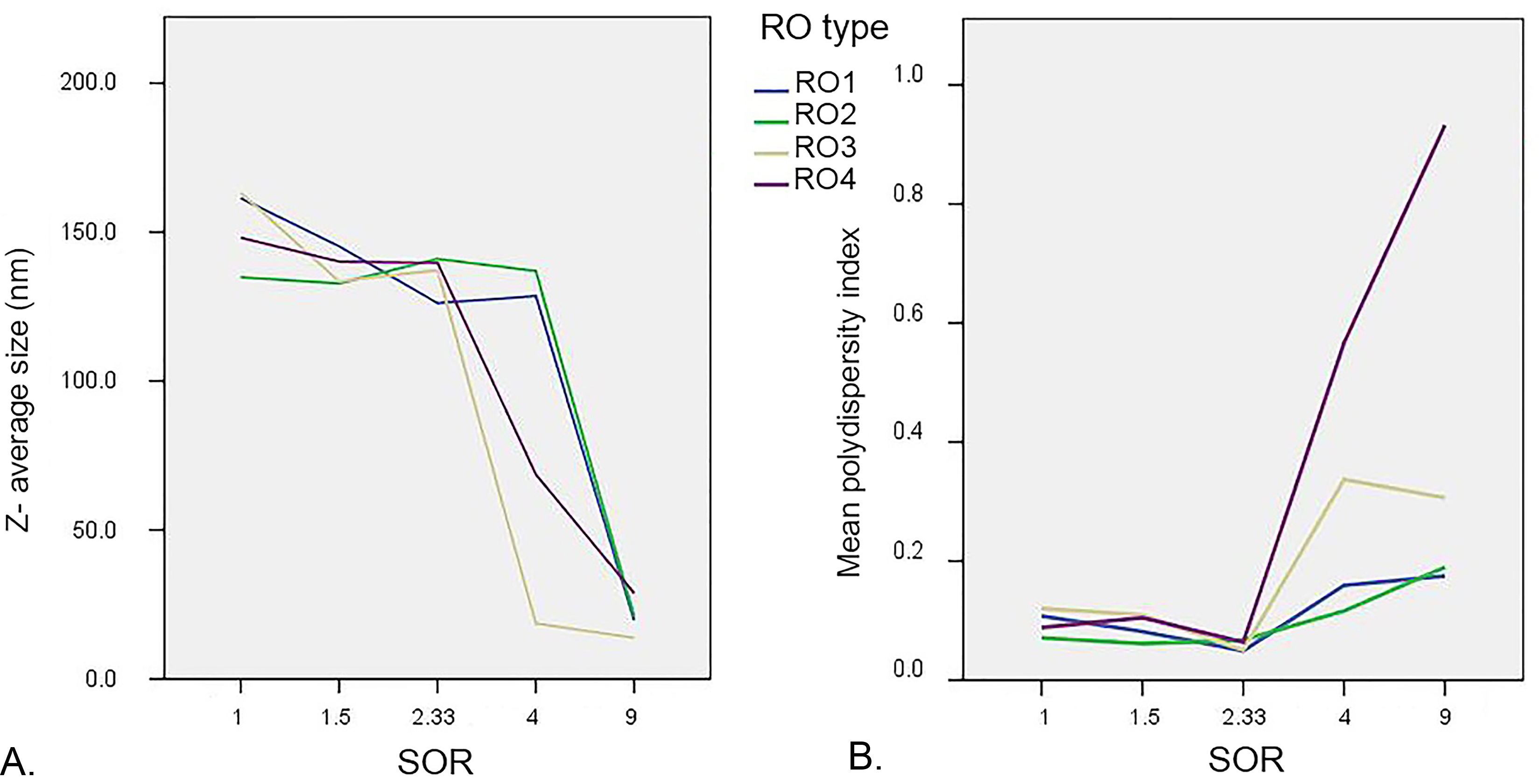

Supplement: S1 Fig — Interactions between the red raspberry seed oil of different type (RO type) and SOR: A. Z-average droplet size as a function of SOR, B. Mean polydispersity index as a function of SOR. (TIF) [file pone.0230993.s003.tif]

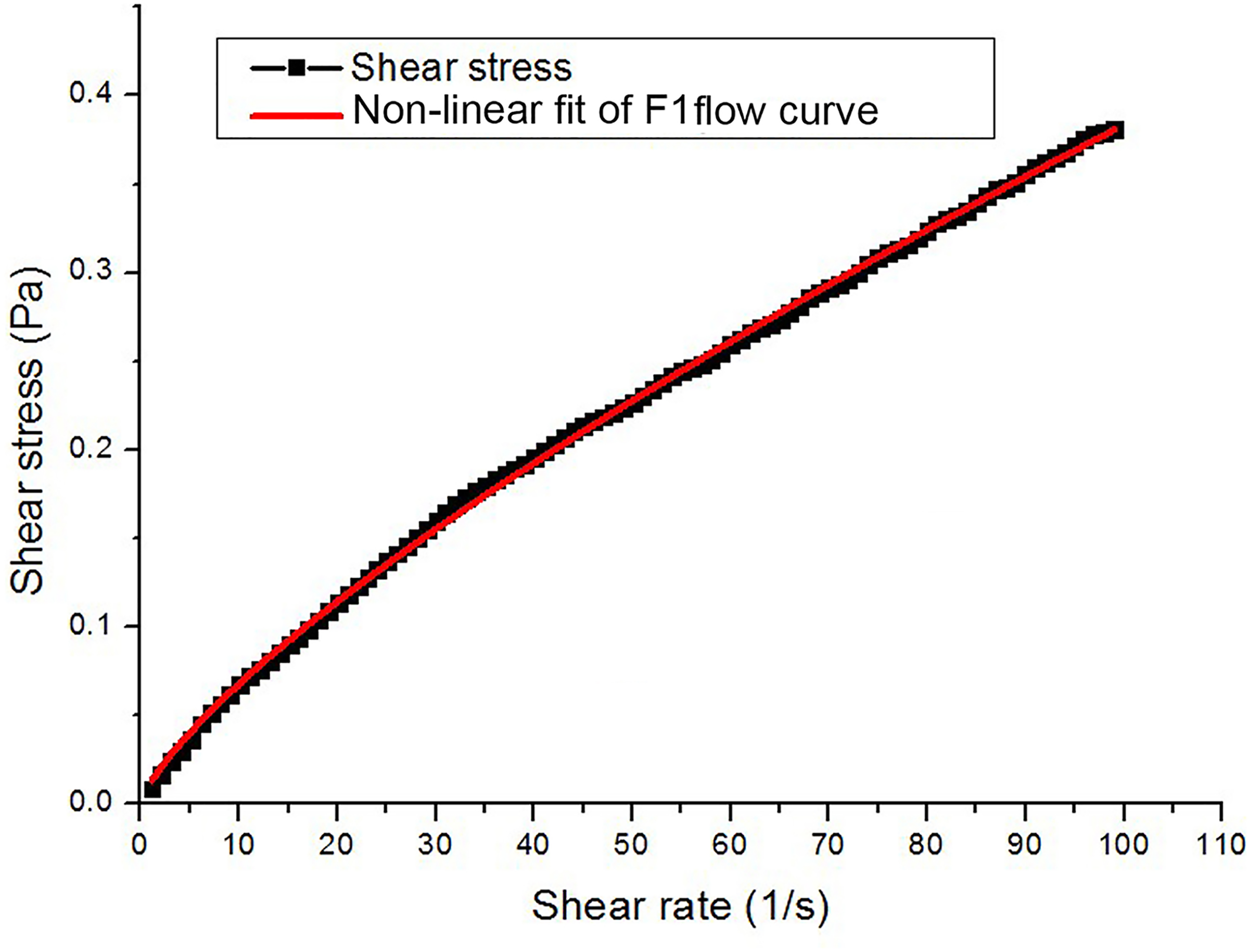

Supplement: S2 Fig — (TIF) [file pone.0230993.s004.tif]

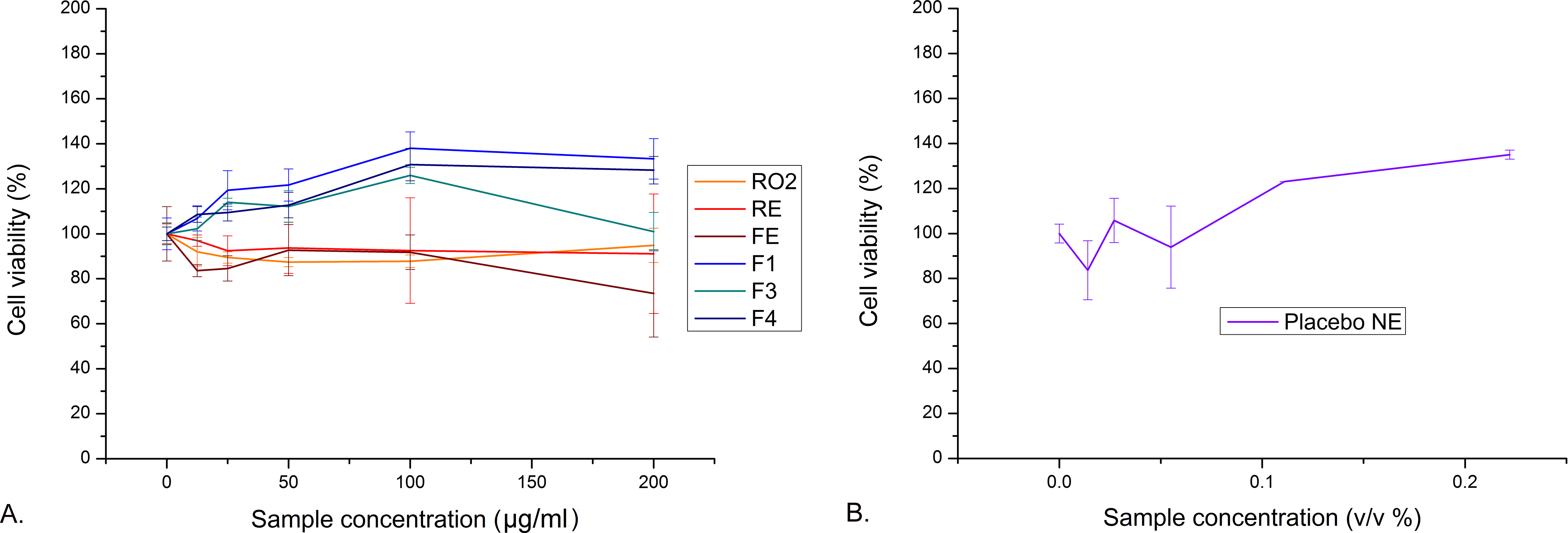

Supplement: S3 Fig — Cell viability assay performed on Fem-X human malignant melanoma cells: (A) Effect of the raw materials and the LE-NE formulations, (B) Effect of the placebo NE. Obtained data represent cell viability % of the cell culture depending on the sample concentration. Each experiment was repeated three times and the results were presented as the mean value ± SD. (TIF) [file pone.0230993.s005.tif]

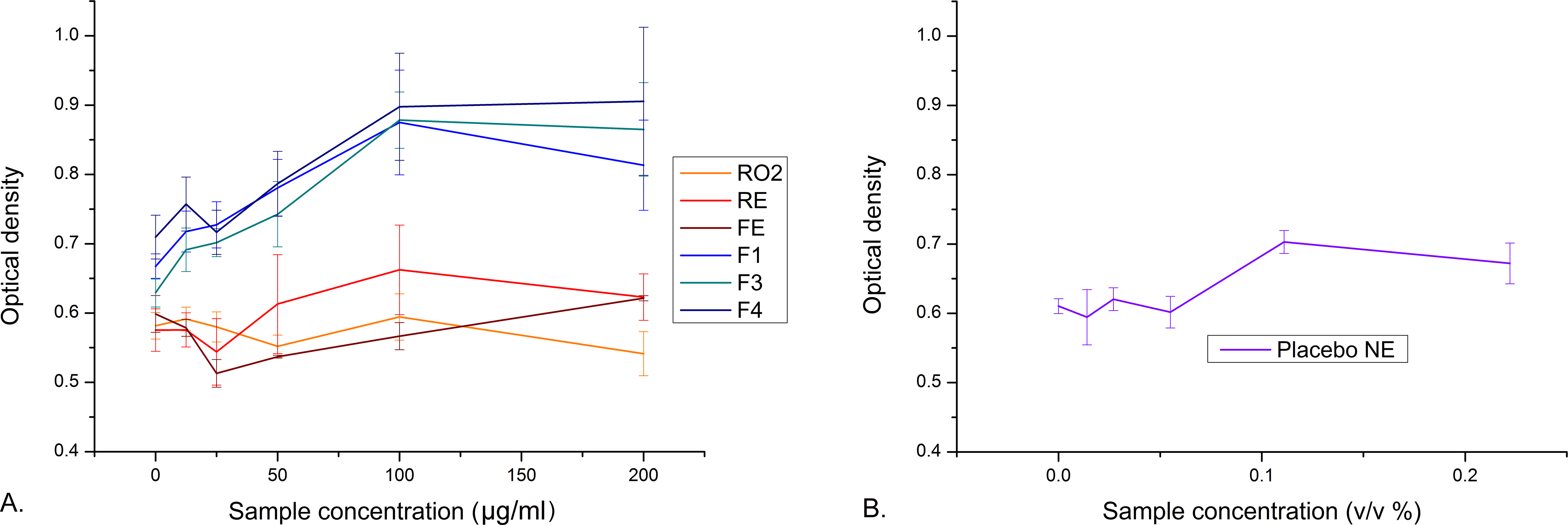

Supplement: S4 Fig — Optical density at 570 nm of the MRC-5 normal human lung fibroblast cells: (A) Effect of the raw materials and the LE-NE formulations, B) Effect of the placebo NE. (TIF) [file pone.0230993.s006.tif]

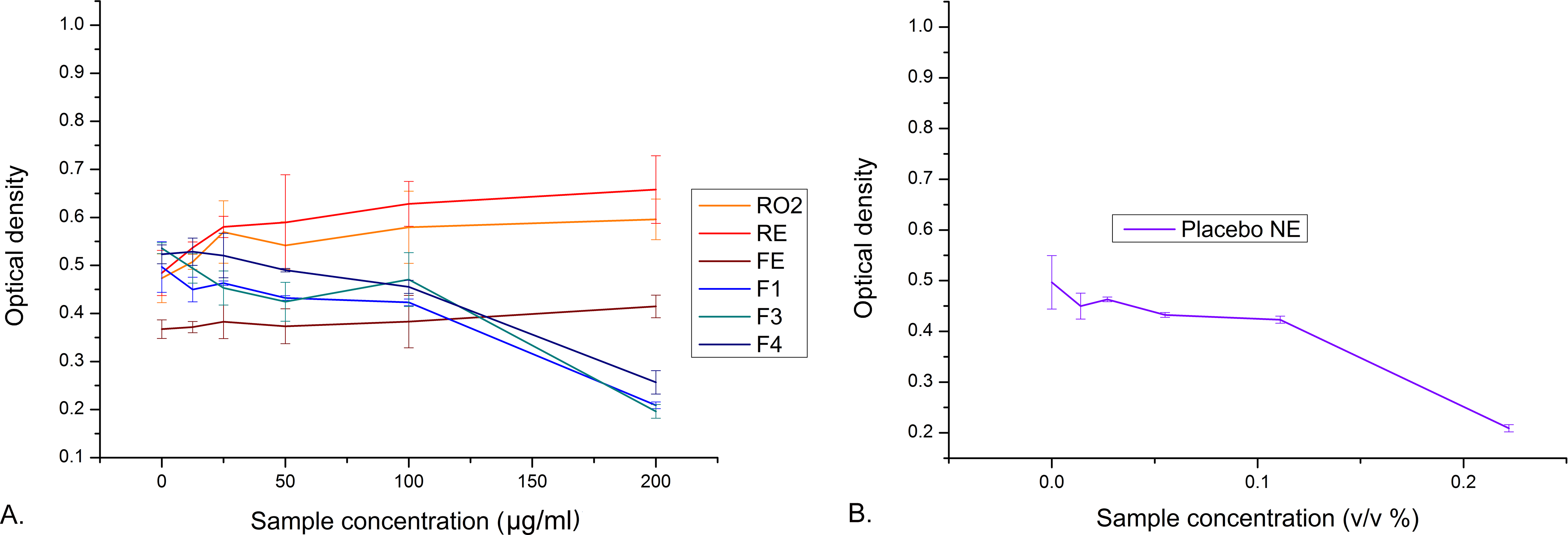

Supplement: S5 Fig — Optical density at 570 nm of the HeLa human adenocarcinoma cells: (A) Effect of the raw materials and the LE-NE formulations, B) Effect of the placebo NE. (TIF) [file pone.0230993.s007.tif]

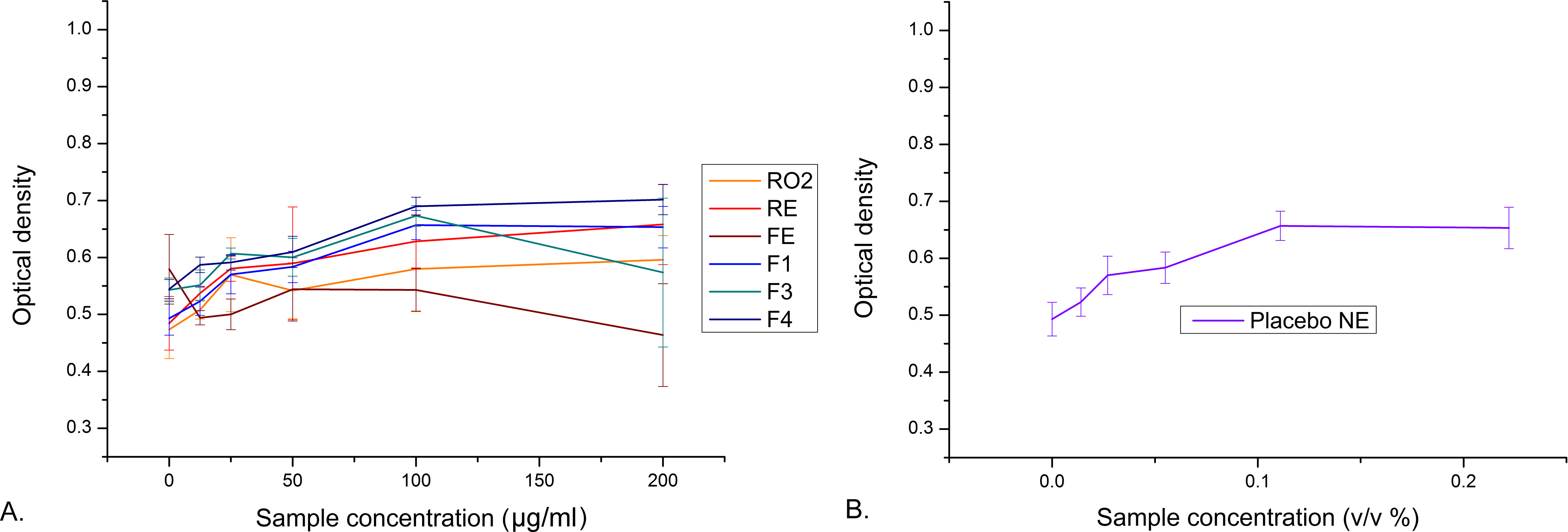

Supplement: S6 Fig — Optical density at 570 nm of the Fem-X human malignant melanoma cells: (A) Effect of the raw materials and the LE-NE formulations, B) Effect of the placebo NE. Obtained data represent optical density of the cell culture depending on the sample concentration. Each experiment was repeated three times and the results were presented as the mean value ± SD. (TIF) [file pone.0230993.s008.tif]
